# Synthetic Lethality of Cohesins with PARPs and Replication Fork Mediators

**DOI:** 10.1371/journal.pgen.1002574

**Published:** 2012-03-08

**Authors:** Jessica L. McLellan, Nigel J. O'Neil, Irene Barrett, Elizabeth Ferree, Derek M. van Pel, Kevin Ushey, Payal Sipahimalani, Jennifer Bryan, Ann M. Rose, Philip Hieter

**Affiliations:** 1 Department of Medical Genetics, University of British Columbia, Vancouver, Canada; 2 Michael Smith Laboratories, University of British Columbia, Vancouver, Canada; 3 Department of Biochemistry, University of British Columbia, Vancouver, Canada; 4 Department of Statistics, University of British Columbia, Vancouver, Canada; University of California Berkeley, Howard Hughes Medical Institute, United States of America

## Abstract

Synthetic lethality has been proposed as a way to leverage the genetic differences found in tumor cells to affect their selective killing. Cohesins, which tether sister chromatids together until anaphase onset, are mutated in a variety of tumor types. The elucidation of synthetic lethal interactions with cohesin mutants therefore identifies potential therapeutic targets. We used a cross-species approach to identify robust negative genetic interactions with cohesin mutants. Utilizing essential and non-essential mutant synthetic genetic arrays in *Saccharomyces cerevisiae*, we screened genome-wide for genetic interactions with hypomorphic mutations in cohesin genes. A somatic cell proliferation assay in *Caenorhabditis elegans* demonstrated that the majority of interactions were conserved. Analysis of the interactions found that cohesin mutants require the function of genes that mediate replication fork progression. Conservation of these interactions between replication fork mediators and cohesin in both yeast and *C. elegans* prompted us to test whether other replication fork mediators not found in the yeast were required for viability in cohesin mutants. PARP1 has roles in the DNA damage response but also in the restart of stalled replication forks. We found that a hypomorphic allele of the *C. elegans SMC1* orthologue, *him-1(e879)*, genetically interacted with mutations in the orthologues of *PAR* metabolism genes resulting in a reduced brood size and somatic cell defects. We then demonstrated that this interaction is conserved in human cells by showing that PARP inhibitors reduce the viability of cultured human cells depleted for cohesin components. This work demonstrates that large-scale genetic interaction screening in yeast can identify clinically relevant genetic interactions and suggests that PARP inhibitors, which are currently undergoing clinical trials as a treatment of homologous recombination-deficient cancers, may be effective in treating cancers that harbor cohesin mutations.

## Author Summary

Synthetic lethality has been proposed as a way to leverage the genetic differences found in tumor cells to affect their selective killing. Many tumor types contain mutations in the cohesin genes. Identifying synthetic lethal genetic interactions with cohesin mutations therefore identifies potential therapeutic targets for these tumors. We have used the simple model organisms yeast and *C. elegans* to screen for synthetic lethal genetic interactions with cohesin mutations. We found that cohesin mutants require the function of proteins that regulate the replication fork for viability. In human cells, Poly-ADP Ribose Polymerases (PARPs) have been shown to repair stalled replication forks and are the target of a class of anti-tumor chemotherapeutics called PARP inhibitors. Based on our finding that cohesin mutants required replication fork regulators and the role of PARP at the replication fork, we tested the effect of PARP inhibitors in human cells with reduced cohesin. We found that PARP inhibitors reduced the viability of cohesin-depleted cells suggesting that PARP inhibitors may be effective for the treatment of tumors containing cohesin mutations. Together our data demonstrate the utility of comprehensive interaction screens in simple organisms to identify clinically relevant genetic interactions.

## Introduction

Defects in cohesin-associated genes are emerging as potential drivers of tumor genomic instability and progression. Mutations in cohesin genes have been identified in several tumor types (Reviewed in [1]). Sequencing of over 200 human orthologs of yeast chromosome instability (CIN) genes from 130 colon tumors found that cohesin genes are mutated in 8% of tumor samples [2]. In a recent study, Solomon *et al.* found the cohesin gene *STAG2* mutated in 21% of Ewing's sarcomas and in 19% of both melanoma and glioblastoma tumors [3]. Furthermore, altered cohesin gene expression, either overexpression or underexpression is characteristic of many tumors [4]–[7]. It has been shown that loss of cohesin subunits induces genomic instability in human cancers and the associated aneuploidy, as is observed in many cell lines with mutations in cohesin, can itself lead to further genomic instability [2], [3], [8]. These observations and the elevated mutational frequency of cohesin in diverse tumor types suggest that cohesin dysfunction may contribute to tumor development and progression.

Cohesins maintain sister chromatid cohesion and screens for defects in sister chromatid cohesion have identified the core cohesin complex, composed of Smc1, Smc3, Scc1 and Scc3, and additional accessory and regulatory proteins [9]. Cohesins form a ring structure that is thought to encircle sister chromatids and physically tether them together until it is cleaved by separase during anaphase [10], [11]. Cohesin proteins contribute to DNA repair and the regulation of gene expression in addition to chromosome segregation (Reviewed in [12]). Although much is known about the function of cohesin in regulating sister chromatid cohesion and DNA damage repair, it is not as yet clear which aspects of cohesin biology might contribute to tumor progression.

One approach to understanding the functional spectrum associated with a gene of interest relies on the identification of genetic interactions with other gene mutations. Negative genetic interactions occur when the double mutant shows a synthetic growth defect manifested as severe slow growth or lethality (synthetic sickness/lethality) when compared to both single mutants. Synthetic sick or lethal interactions with genes mutated in cancer can identify potential therapeutic targets [13], [14]. A clinically relevant example of a synthetic lethal (SL) genetic interaction is the SL interaction between mutations in breast cancer susceptibility genes *BRCA1* or *BRCA2* and loss of function of the Poly-ADP Ribose Polymerases (PARP). Two groups found that *BRCA1*- and *BRCA2*-defective cells are sensitive to knockdown of PARP or chemical inhibition of PARP activity [15], [16]. This has lead to the development of PARP inhibitors as chemotherapeutics. PARP inhibitors are being evaluated in Phase II clinical trials for use in homologous recombination (HR) deficient breast and ovarian tumors [17]–[20].

In addition to identifying synthetic lethal interactions that could represent potential drug targets, comprehensive genetic interaction networks can also lead to new functional insights [21]. Mapping global genetic interactions in human cells is feasible but techniques lag behind those currently available in budding yeast. Synthetic genetic array (SGA) is a large-scale genetic interaction screening approach in yeast that facilitates the collection and analysis of positive and negative genetic interaction data [22]–[24]. The use of yeast as a model organism to identify conserved genetic interactions with potential cancer therapeutic value has proven effective [14]. The inclusion of the metazoan animal model, *C. elegans*, in the genetic interaction testing pipeline can also contribute new insights as nematodes have a gene complement more akin to humans and contain several cancer-relevant genes not found in yeast, such as *BRCA1*, *BRCA2*, *TP53* and the family of poly(ADP)-ribose polymerases (*PARPs*) [25]–[28]. Furthermore, *C. elegans* mutants and double mutants also present informative phenotypes, such as apoptotic defects, cell cycle checkpoint dysfunction, and chromosome loss, in the context of a multicellular animal model, which can lead to a better understanding of the biological processes affected by specific genetic interactions [29]. The large number of genetic interactions that can be identified by comprehensive genetic interaction screens such as SGA can identify key processes or pathways that when disrupted result in synthetic lethality. These pathways could be targeted for SL therapeutic intervention even if the specific genes are not well conserved from the yeast to humans.

In this study, we performed digenic SGA screens in *S. cerevisiae* using three hypomorphic cohesin mutations to identify common processes required for survival when cohesin is mutated. Interactions were tested for conservation in *C. elegans* using an assay for defects in somatic cell proliferation [30]. We found that proteins mediating replication fork progression and stability are required in cohesin mutants of both *S. cerevisiae* and *C. elegans*. Based on these findings we predicted that other mediators of replication fork stability not conserved in yeast, such as PARP, would be required for viability in higher eukaryotic cells with mutations in cohesin. To test this prediction, we expanded the screen in *C. elegans* to include PAR metabolism (*pme*) mutations and found that *pme* mutants genetically interact with *him-1/SMC1* in *C. elegans*. We found that this genetic interaction was conserved in cultured human cells and small molecule PARP inhibitors, currently being evaluated in clinical trials, were effective in inhibiting growth in cohesin depleted cultured human cells. Beginning with systematic screens in a simple model eukaryote this study identifies conserved and clinically relevant genetic interactions between cohesin and replication fork modulators including the chemotherapeutic target PARP.

## Results

### Systematic quantitative analysis of cohesin genetic interactions

Synthetic genetic array (SGA) technology was used to screen temperature sensitive (ts) alleles of two cohesin components (*smc1-259, scc1-73*) and one cohesin loader (*scc2-4*) against ∼95% of genes in *S. cerevisiae* as represented by non-essential gene deletions [31], ts [32] or decreased abundance by mRNA perturbation (DAmP) [33] alleles. All three cohesin alleles have mutations in similar regions as those identified in colon tumors (*SMC1, SCC2*) or in the Catalogue of Somatic Mutations in Cancer (COSMIC) (*SCC1*) (Table 1, Figure S1).

**Table 1:** Mutations in cohesin genes seen in tumors.

Human Gene(Yeast Gene)	Mutations reported in CIN colon tumors	Cancer mutations reported in COSMIC*; cancer type
*SMC1L1 (SMC1)*	F369L (1186C>T)R434W (1300C>T)I560M (1680 C>G)I1186V (3556G>A)	
*CSPG6 (SMC3)*	R879X (2635C>T)	A44V (131C>T); CNS
*RAD21 (SCC1)*		Q474stop (1420C>T); lungE498K (1492G>A); skin
*STAG3 (SCC3)*	I795T (24117T>C)	F660L (1980C>A); lung
*NIPBL (SCC2)*	R479STOP (1435C>T)Frameshift after aa 992Q554STOP (1660C>T)M1793K (5378T>A)	E1674K (4939G>A); breastS349C (1045A>T); lungDeletion, Frameshift after aa 2241; lung

*: Silent mutations not reported in this table. Amino acid: aa.

**Figure 1:**
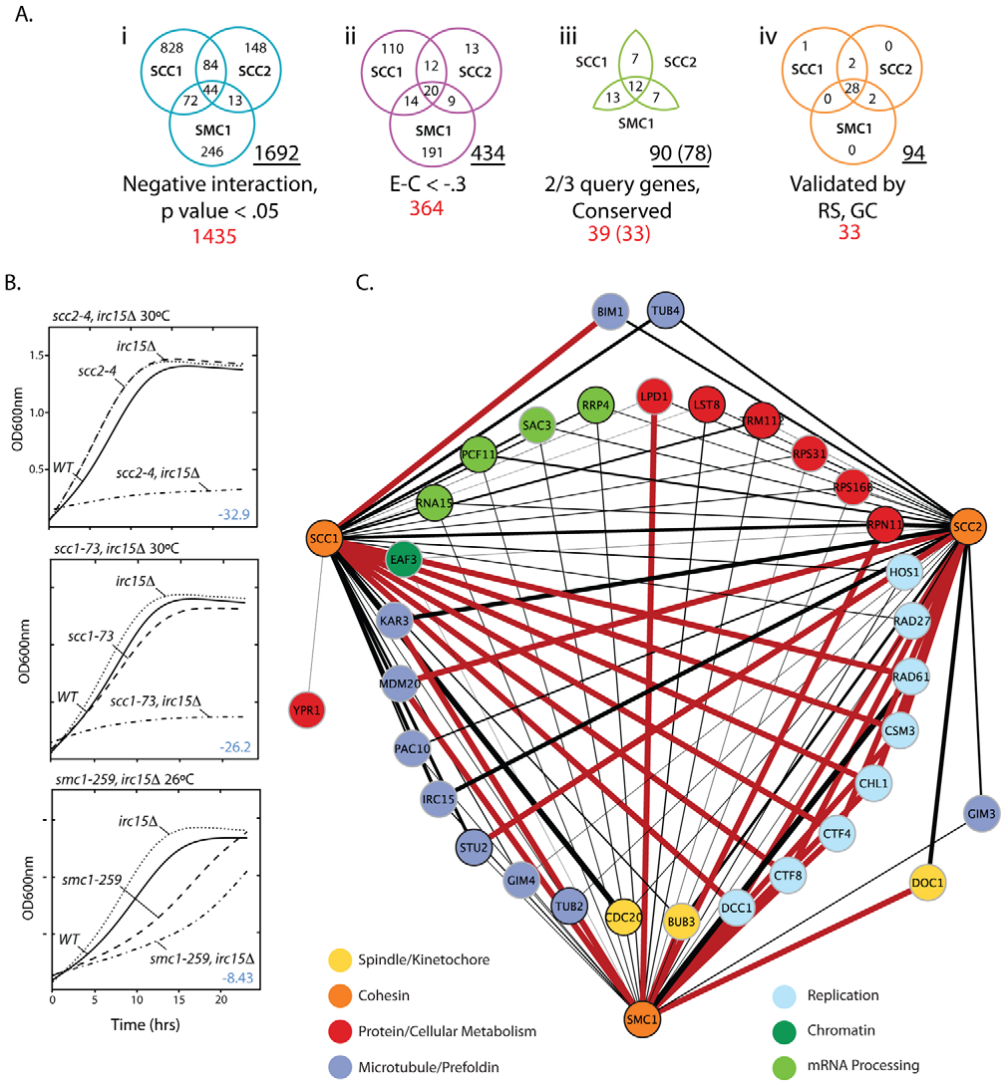
*S. cerevisiae* cohesin genetic interaction network. A) Venn diagrams depicting how SGA data was filtered. i. Interactions that had a negative interaction value and were statistically significant (p-value<0.05). ii. Interactions that had a relatively weak interaction score (Experimental value – Control value>−.3) were eliminated to enrich for biologically significant interactions. iii. Genes were eliminated if they failed to interact with ≥2 of the cohesin query genes and if they did not have a human homolog (Table S3). iv. Summary of the final network after random spore and growth curve retesting and validation. The total number of interactions in a given Venn diagram is underlined. The total number of genes is shown in red. The numbers in brackets indicate interacting genes remaining after removal of 6 genes for technical reasons. B) Representative subset of growth curve data. The T-stat, which takes into account the interaction magnitude and statistical significance, is shown in blue. All growth curve data can be found in Figures S3, S4, S5, S6 and Tables S1, S2, S3. C) Expanded view of the final network summarized in A iv. Red lines indicate SL interactions and black lines represent SS interactions. The black line thickness represents interaction strength.

The interaction data was sequentially filtered using several criteria to increase quality and focus. Only negative genetic interactions with a p-value less than 0.05 and a large interaction magnitude (E-C value less than −0.3, see Methods) were considered. Filtering based on magnitude enriched for interactions that cause a severe fitness defect when compared to the single mutant. To reduce false positives and increase the potential of identifying drug targets that interact with mutations in multiple cohesin subunits, we eliminated genes that interacted with only a single cohesin query gene, leaving 55 genes (Figure S2). Finally, to focus on interactions that may have relevance to the biology of cohesins and cancer, we eliminated genes that did not have an obvious human homolog (see Figure 1A, Table S1). Using these criteria, 39 of 55 genes (71%), defining 90 putative negative genetic interactions, had an identifiable homolog in humans (Table S1). Six genes were removed from further analysis for technical reasons, leaving 33 genes comprising 78 genetic interactions.

To validate this subset of interactions identified in the primary screen, 99 double heterozygous diploids (33 genetic interaction genes by 3 cohesin query genes) were reconstructed, the specific gene deletions confirmed by DNA analysis, and double mutant phenotypes retested by random spore analysis. To assess whether growth defects were greater than additive, growth curve analysis was performed on all viable double mutants (Figure 1B; Figures S3, S4, S5, S6, S7; Tables S2, S3, S4). Random spore and growth curve analyses achieved several goals; 1) Reduced the false positive rate by removing genes with an incorrect well address in the high throughput arrays (8% of hits), 2) Eliminated condition artifacts by ensuring that genetic interactions were reproducible under the same drug selective conditions as SGA (random spore) and in rich medium (growth curve), 3) Yielded an additional quantitative measure of each synthetic sick interaction, and 4) Identified additional true positives not identified under SGA conditions.

During subsequent testing 4 out of 78 (5%) interactions identified by SGA did not result in a negative genetic interaction and an additional 20 interactions not observed by SGA were identified for a total of 94 negative genetic interactions with the three cohesin query genes (Figure 1C). 29 (31%) of these interactions involve essential genes, highlighting the importance of screening against essential gene collections.

### Negative genetic interactions with cohesin are conserved in *C. elegans*

**Figure 2:**
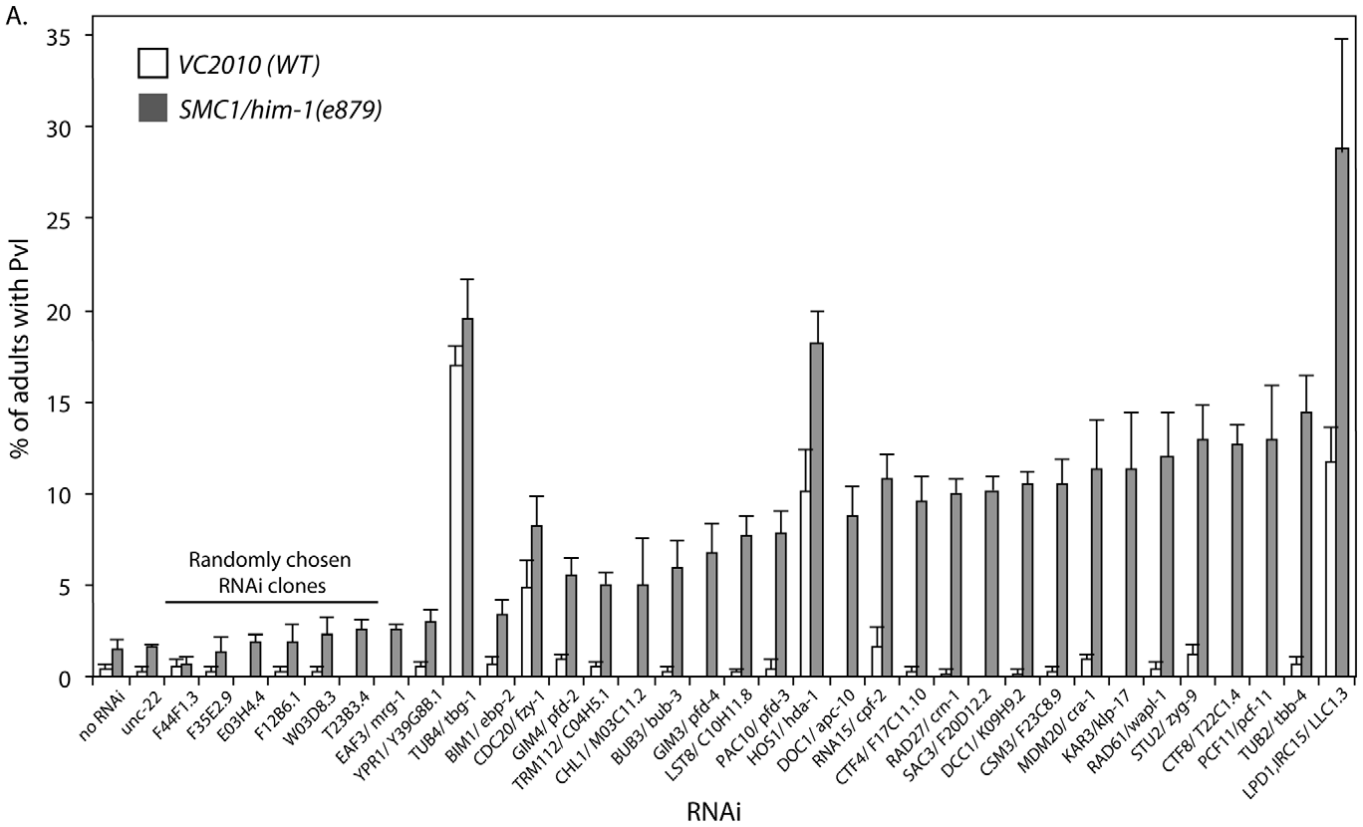
*C. elegans* genetic interactions. Graph depicting the frequency of worms with a protruding vulva (Pvl) when *VC2010* (WT) and *SMC1/him-1(e879)* strains are treated with various RNAi constructs. The RNAis tested targeted the *C. elegans* homologs of genes that interact with one of the cohesin query genes in the *S. cerevisiae* validated network (Figure 1C). Homologs with associated e-value BLAST scores can be found in Table S1. Interactions are ranked by the difference between the frequency of Pvl in VC2010 and *him-1* strains. The predicted value is the sum of both the him-1 and N2 background Pvl frequencies and the effect of the RNAi on WT. *unc-22* and 6 randomly chosen RNAi clones from chromosome 1 are included as negative controls. Error bars represent SEM.

One of our goals is to identify interactions that are conserved in mammalian cells and thus relevant to the development of therapeutics. We hypothesized that interactions that are conserved among eukaryotes are more likely to be conserved in higher animals and therefore we tested the validated *S. cerevisiae* interactions in the model metazoan, *C. elegans*. We used a visual screen that monitors defects in development of the *C. elegans* vulva to identify synthetic genetic interactions that resulted in defects in somatic cell proliferation [30]. These vulval cell divisions occur late in nematode development [34], so perturbations do not affect organismal viability, which allows us to screen for interactions using gene mutations or RNA interference (RNAi) knockdowns that are potentially embryonic lethal. We tested the *C. elegans* homolog of all genes that had a verified genetic interaction with one of the cohesin genes in *S. cerevisiae* and for which there was an RNAi construct available (Table S1). Cohesins are essential genes, so we used a viable hypomorphic mutation in the *C. elegans SMC1* ortholog, *him-1(e879)*, and treated these worms with RNAi by feeding. Adult worms were bleached to obtain embryos that hatched into onto plates seeded with bacteria expressing different dsRNA constructs. When the embryos hatch into L1 larva they eat the dsRNA-expressing bacteria, which initiates systemic RNAi knockdown. An increased frequency of defects in the mutant treated with RNAi, compared to the predicted additive effect for the mutant and RNAi separately, is indicative of a genetic interaction. We observed a clear increase over the predicted additive frequency of Pvl in 23/28 (82%) interactions tested (Figure 2) demonstrating that most interactions are conserved between *S. cerevisiae* and *C. elegans*.

### Analysis of cohesin network sub-groups

Each gene in the network was assigned to one of several broad functional groups based on gene descriptions reported in the *Saccharomyces* Genome Database (SGD). These groups were the spindle, microtubules and kinetochore (39%), mRNA processing (12%), replication factors (24%), and a group that contains genes involved in general cell metabolism (24%).

One hypothesis for a genetic interaction is that two mutations that cause the same phenotype, when combined cause a cumulative phenotype that breaches the tolerance level of the cell. One phenotype of cohesin mutants is increased CIN and we therefore investigated whether the interactors also cause CIN when mutated. For this analysis we used information collected from SGD and a recently performed screen for CIN in essential genes [35] (Table S5). We found most of the genes, except those in the metabolism group, cause CIN as measured by a variety of assays such as chromosome transmission fidelity (CTF) and gross chromosomal rearrangements (GCR) in yeast [35].

### Cohesin mutations are synthetic lethal with mutations in replication fork mediators

**Figure 3:**
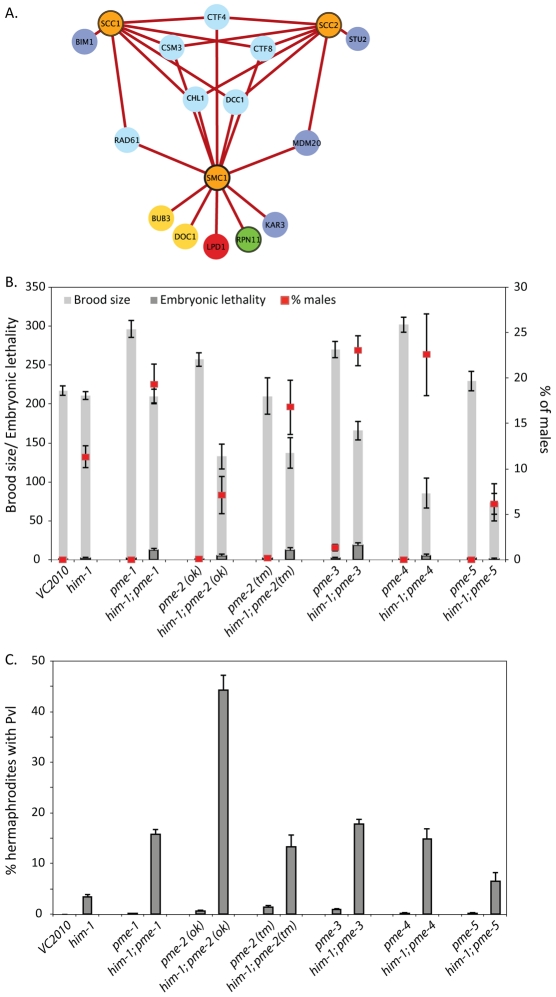
Sub-optimal cohesin requires replication fork mediators. A) The validated *S. cerevisiae* cohesion network was filtered to include only SL interactions. Gene nodes are colored according to the legend in Figure 1C. B) Brood size (total number of eggs laid), embryonic lethality, and percentage of male progeny and C) Frequency of Pvl in the indicated single and double mutants. Error bars represent SEM.

To probe the identified interactions further, we profiled the interactions based on strength by filtering the network to include only SL interactions. We hypothesized that these interactions would indicate the most critical processes when cohesin genes are disrupted (Figure 3A). This analysis revealed five genes specifically involved in replication fork progression and stability that were SL with all three cohesin alleles tested. Ctf8p and Dcc1p are components of the alternative replication factor C Ctf18 clamp loader (altRFC^CTF18^) that controls the speed and restart activity of the replication fork [36]. Rad61p acts with Pds5p to bind cohesin and regulates its association with chromatin [37], [38]. Chl1p is a DNA helicase that interacts with *CTF18* and physically with Eco1p [39]. Ctf4p (human AND1) has a role in coupling the Mmc2-7p helicase replication progression complex to DNA polymerase α [40]. Csm3p functions in a complex with Tof1p and Mrc1p to control stable pausing of the replication fork [41], [42]. We collectively call these genes replication fork mediators. The SL interaction of replication fork mediators with multiple cohesin mutants suggests that cohesin mutations sensitize cells to perturbations of the replication fork.

To investigate the biology underlying the interaction between cohesin and replication fork mediator mutations the frequency of apoptosis in the *C. elegans* germline was analyzed. In the *C. elegans* germ line, nuclei with DNA damage are removed by apoptosis and increased germline apoptosis can be indicative of increased DNA damage [43]. Apoptosis levels were quantified in the germline of the *him-1(e879)* mutant and were found to be elevated above that of wild type (Figure S8). When *him-1* mutant animals were treated with RNAi against *csm-3*, *rad-61*, *ctf-8*, *ctf-4* and *dcc-1* an increase in apoptosis was detected. Apoptotic bodies in these cases were typically found in large clusters, rather than being distributed throughout the pachytene region, reminiscent of irradiated animals (Figure S8). These results suggested that the defects in both HIM-1 and replication fork mediator function leads to DNA damage that results in increased apoptosis.

We have shown that cohesin mutations are synthetic lethal with mutations in genes that mediate replication fork progression (Figure 1, Figure 2, Figure S8) but are not synthetic lethal with any DNA repair mutants (Table S6). These results suggest that the cohesin mutations lead to replication fork progression defects but not directly to DNA damage. This is further supported by the observation that the yeast cohesin mutations do not result in Rad52p foci accumulation, which are indicative of HR repair intermediates [44]. Additionally, elimination of the HR repair pathway by deletion of *RAD51* in replication fork mediator-cohesin double mutants did not rescue lethality, suggesting that toxic recombination intermediates are not the cause for lethality in a cohesin mutant background (Figure S9). These data suggest that the interaction between cohesin mutants and fork mediators is intimately tied to the regulation of replication progression and led us to further investigate this relationship.

### *him-1* genetically interacts with the PARP pathway in *C. elegans*

The finding that replication fork mediator mutations are synthetic lethal with hypomorphic mutations in cohesin led us to hypothesize that inhibitors of replication fork stability could result in specific killing of tumors containing cohesin mutations. At present there are no small molecule inhibitors of the replication fork mediators identified in our SGA screen. However, in higher eukaryotes there are additional factors that protect and regulate the replication fork. An early mediator of replication fork stability is the family of Poly (ADP-ribose) polymerases (PARPs). PARPs have been shown to localize to stalled forks and mediate restart [45]. Furthermore, there are effective small molecule inhibitors of PARP and although PARPs are not present in yeast they are present in *C. elegans*. To assess whether PARP metabolism plays a role in maintaining viability in a cohesin mutant, we made double mutants with *him-1(e879)* and the five PAR metabolism enzyme (*pme*) genes in *C. elegans*. *pme-1* and *pme-2* are the *C. elegans* orthologs of *PARP1* and *PARP2*, respectively [28]. *pme-3* and *pme-4* are homologs of Poly (ADP-ribose) glycohydrolase (*PARG*), which depolymerizes ADP-ribose polymers into monomeric ADP-ribose units [46]. *pme-5* is the *C. elegans* ortholog of *PARP5*, which is also known as *TANKYRASE* [47]. All *him-1; pme* double mutants exhibit decreased brood sizes and an increase in the frequency of arrested embryos, suggesting that *him-1* interacts with all identified members of the PAR metabolism family in *C. elegans* (Figure 3B). Strikingly, *him-1; pme-2(ok344)* double mutants had a very high frequency of protruding vulva phenotype (Figure 3C) indicative of somatic cell proliferation defects.

### PARP inhibition reduces the viability of *SMC1* depleted HCT116 cells

**Figure 4:**
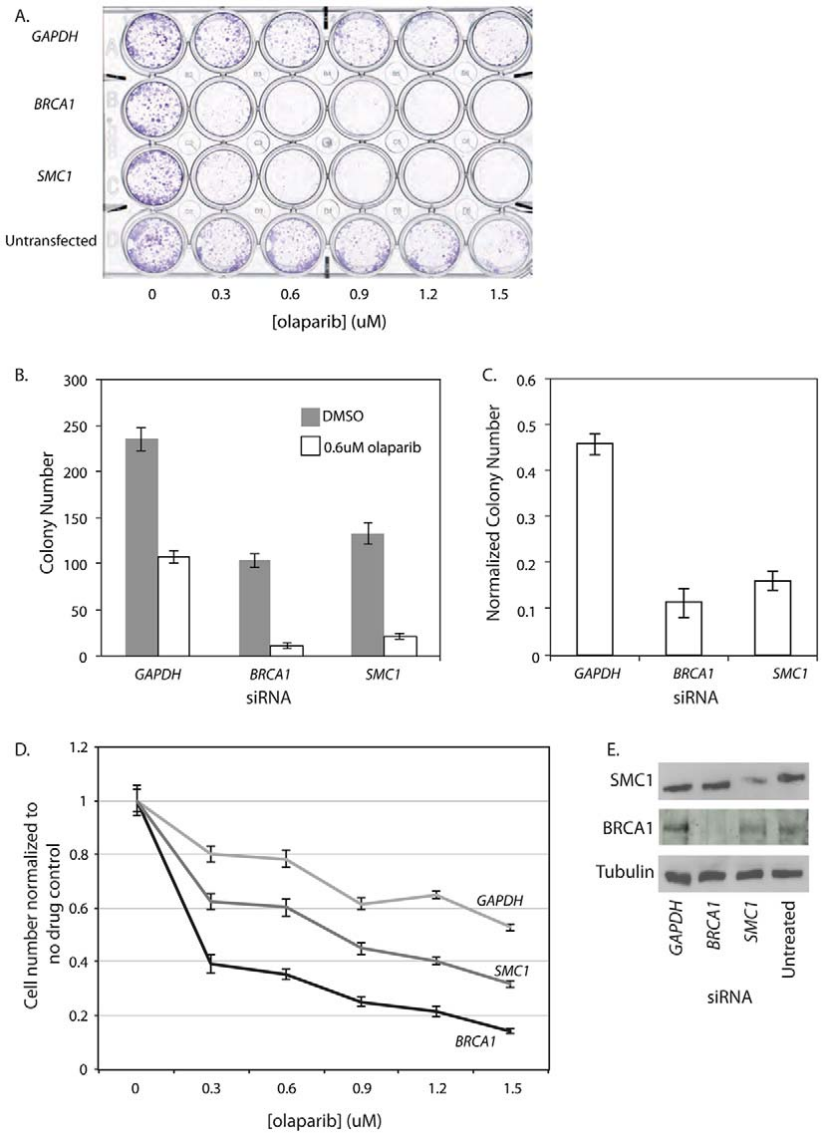
*SMC1* siRNA treated human cells are sensitive to the PARP inhibitor olaparib. All experiments were performed with HCT116, a near diploid, colon cancer derived cell line that is MMR deficient. In all panels HCT116 cells are treated with siRNA targeting *GAPDH*, *BRCA1* or *SMC1* or untreated (in indicated). A) 24 well plate clonagenic survival assay with HCT116 siRNA treated cells exposed to olaparib concentrations up to 1.5 µM. B) 10 cm dish clonagenic survival assay looking at the number of colonies after 10 days in the presence of 0.6 µM olaparib. C) Normalized colony numbers from the clonagenic assay. D) HC-DIM counting Hoescht positive nuclei of siRNA treated cells after 3 days of olaparib exposure. E) Western blot of samples collected from the 10 cm survival assay (B). Error bars represent SEM.

The strong negative genetic interactions in *C. elegans* between a hypomorphic cohesin mutation and the *pme* mutations prompted us to test whether this interaction is conserved in human cells using the colon cancer-derived, near diploid cell line, HCT116 [48]. We used an early generation PARP inhibitor, benzamide [49], to inhibit PARP function in a panel of HCT116 cells treated individually with siRNA against several cohesin genes (*SMC1*, *SMC3*, *SCC2/NIPBL*, *SCC1/RAD21*, *SCC3/STAG1* and *SCC3/STAG3*). High content digital imaging microscopy (HC-DIM) was used to count Hoescht stained nuclei. Although HC-DIM is not necessary to count nuclei it allows more nuclei to be counted and more technical replicates to be performed in a timely manner. This preliminary assay suggested that HCT116 cells depleted of cohesin were sensitive to PARP inhibition (Figure S10). We further investigated this interaction using a more specific, third generation PARP inhibitor, olaparib, which has been evaluated in phase II clinical trials in the treatment of HR deficient breast and ovarian cancers [17], [19], [20]. *BRCA1* siRNA treated cells were used as a positive control for PARP inhibition as *BRCA1*-deficient cells are highly sensitive to PARP inhibitors [15], [16]. Accordingly, we found *BRCA1* siRNA treated cells were very sensitive to olaparib (Figure 4). We also found by visual inspection that *SMC1* siRNA treated cells exhibited reduced cell proliferation in response to a range of olaparib concentrations using a 24 well plate survival assay whereas untreated and *GAPDH* siRNA treated cells appeared only mildly affected (Figure 4A). We quantified the specific sensitivity using an expanded 10 cm dish survival assay where cells treated with siRNAs were continuously exposed to 0.6 µM olaparib and colonies were stained and counted after a 10 day period (Figure 4B, 4C, 4E). We also saw evidence that HCT116 *SMC1* knockdown cells exposed to olaparib exhibited proliferation defects using HC-DIM. Overall, we observed a significant dose dependent decrease in cell number in the *BRCA1* and *SMC1* treated cells as compared to *GAPDH* (Figure 4D).

### PARP inhibition reduces viability in HTB-38, a human cell line with low parylation levels

**Figure 5:**
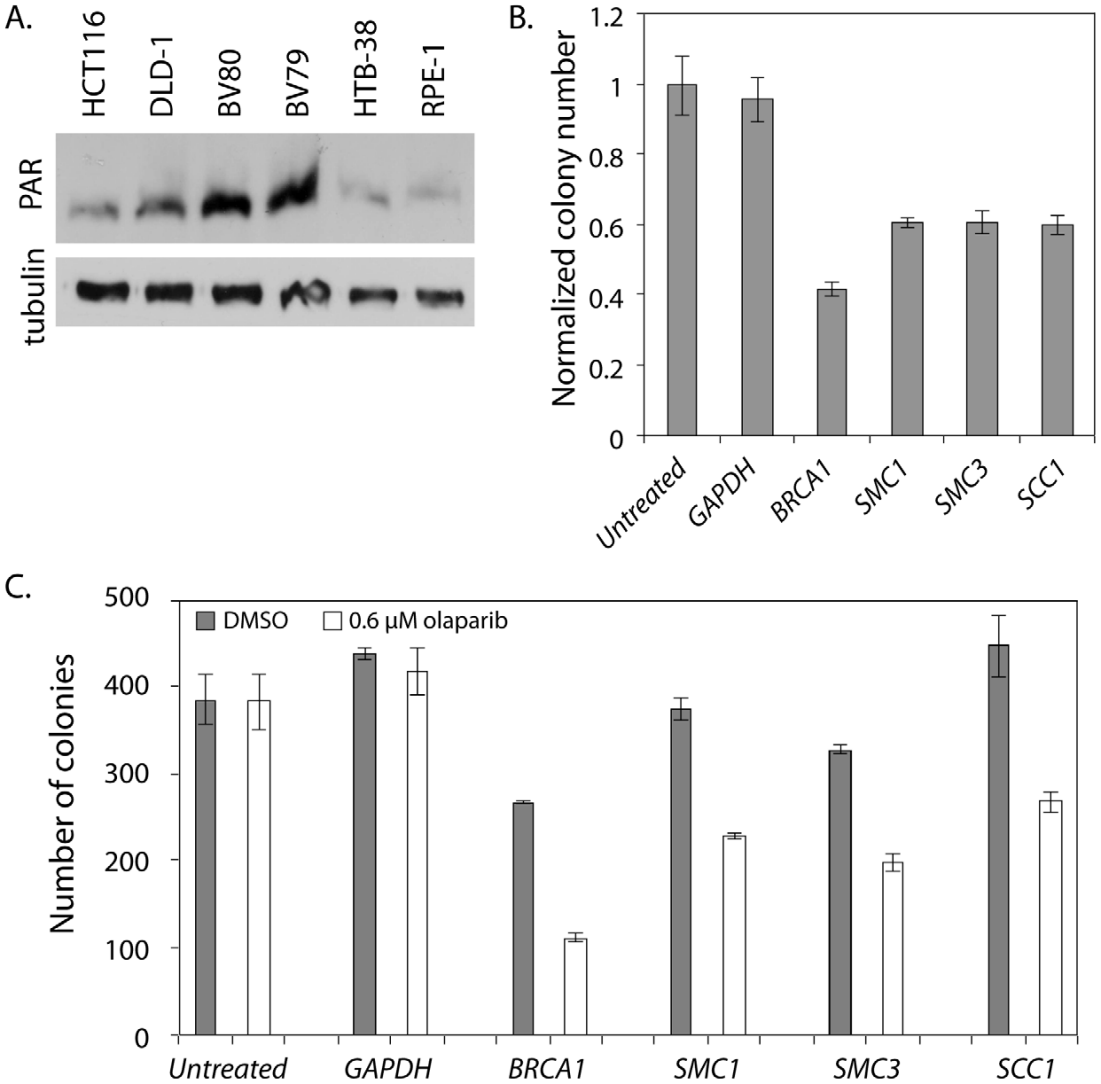
*SMC1* and *PARP* genetically interact in HTB-38 human cells. All experiments were performed with HTB-38, a near triploid colon cancer derived cell line that is MMR proficient. A) Western blot stained with anti-PAR. BV80 is MLH1−/− and is the matched line to BV79 which is MLH1+/−. RPE-1 was used as a control cell line for measuring PAR levels. RPE-1 is an hTERT immortalized retina epithelial cell line. B) Normalized colony numbers for HTB-38 cells treated with the indicated siRNA and exposed to 0.6 µM olaparib. C) Raw colony numbers for the clonagenic assay shown in B. Error bars represent SEM.

We were concerned that the effect of PARP inhibition was complicated by the fact that HCT116 cells have a high level of endogenous PARP activity due to deficiencies in mismatch repair (MMR) [50], [51]. Consistent with previous reports, we observed relatively high PAR levels, which are indicative of PARP activity, in untreated HCT116 cells (Figure 5A). We also found that HCT116 cells treated with 0.6 µM olaparib showed a 54% decrease in viability as measured by a colony forming assay. To rule out the confounding effects of elevated PAR in the HCT116 cell line, we repeated the colony forming assay, with and without olaparib treatment, on a second colon cancer-derived cell line that was MMR-proficient, HTB-38. First, we confirmed that, as previously reported, HTB-38 cell lines did not exhibit increased PAR levels (Figure 5A). We found that *GAPDH* and untransfected HTB-38 cells were insensitive to olaparib (Figure 5B, 5C) unlike the HCT116 cells that showed mild sensitivity to olaparib (Figure 4B). However, olaparib treatment decreased viability in the HTB-38 cells treated with siRNA targeting either *BRCA1* or *SMC1*, confirming the sensitivity to PARP inhibitors of *SMC1*-depleted cells. To determine whether the sensitivity to PARP inhibition was limited to *SMC1* or was more general, extending to defects in other cohesin components, we treated cells with siRNA targeting the cohesin components *SMC3* or *RAD21/SCC1*. We observed growth defects of similar strengths among all cohesin subunits tested. These experiments demonstrate that cells with cohesin defects are sensitive to PARP inhibition.

## Discussion

Cohesin dysfunction appears to have a significant role in the formation and progression of tumors (Reviewed in [1]). Here we show that replication fork stability genes are required for viability in cohesin mutants across species. Using data from yeast SGA analyses we were able to predict synthetic lethal interactions in human cells between cohesin mutations and PARP inhibitors, even though PARP is not present in yeast, demonstrating the power of large-scale genetic interaction screens for synthetic lethality with genes mutated in tumors.

### The spectrum of cohesin genetic interactions

SL interactions in model organisms can identify candidate genes or pathways that can be targeted for inhibition leading to specific killing of tumor cells with specific mutations [13]. We used SGA technology in yeast to generate a network of negative genetic interactions, using hypomorphic mutations in cohesin as query genes, with the aim of elucidating the genetic pathways needed for survival when cohesin function is compromised. By overlaying the results from three separate SGA screens, using two cohesin core components (*SMC1* and *SCC1*) and one cohesin loader (*SCC2*) as queries, we identified common interactions with compromised cohesin rather than those that were allele or component-specific. While the screens found scores of single negative interactions with each of the three query mutants (Figure 1A), the goal of this study was to identify interactions that are more likely to be SL with the wide-range of cohesin-associated mutations observed in human tumors. Negative genetic interactions specific to only one of the three cohesin mutants may reveal specific aspects of cohesin components and these interactions warrant further investigation.

The filtered *S. cerevisiae* interaction set identifies many processes that would be predicted to interact with cohesin dysfunction. For example, mutations affecting the mitotic spindle, kinetochore and microtubules, including prefoldin, were found to result in synthetic growth defects with at least two of the three query mutants. Given the role of cohesin in regulating chromosome segregation leading to the anaphase transition [9], mutations affecting the spindle or kinetochore would be predicted to interact with cohesin mutations. Other interactions that do not appear to be related to cohesin function may represent more general effects on cell viability; for example ten interactions were identified with components of mRNA processing and the translation machinery. In these cases, further work will be needed to ascertain whether this is a general synergistic effect on viability or whether the interaction results in a specific defect.

When interactions were filtered based on strength, keeping only those interactions that were SL, the predominant genes were those involved in mediating replication fork progression. These included two of the three genes in the alternative RFC^CTF18^, the replisome components *CSM3* and *CTF4*, the replication and cohesion-associated helicase *CHL1*, and the cohesin regulator *RAD61*. All of these interactors are known to mediate replication fork stability and progression [36]–[42], [52]–[54]. Furthermore, mutations affecting these genes also result in chromosome cohesion defects [55], thereby linking replication fork stability and sister chromatid cohesion. The SL interactions are not specific to mutations that accelerate or impair replication fork progression. In fact, the Alt-RFC^CTF18^, which promotes replication fork progression, does so by regulating the acetylation of Smc3p [36], which in turn inhibits the association of Smc3p and Rad61p [52], [53]. In contrast to the Alt-RFC^CTF18^, Rad61p binding to Smc3p slows replication fork progression [36]. Rad61p is known to destabilize the association between cohesin and chromosomes [38]. The requirement for *RAD61* in the cohesin mutants is particularly interesting given the correlation of elevated expression of the human *RAD61* ortholog, WAPL, with certain cancers. Its importance in maintaining the viability of tumor cells was demonstrated by the fact that knockdown of WAPL in cervical cancer cell lines resulted in cell death [4]. Similarly, we observe synthetic lethality when we knockout *RAD61* in the cohesin mutants, thereby demonstrating that Rad61p function is needed when cohesin function is compromised.

The synthetic lethality of the cohesin mutations with replication fork mediator mutations suggested that the replisome must be stabilized when cohesin is mutated to ensure proper progression. Interestingly, cohesin mutants did not show negative genetic interactions with DNA repair genes such as the *RAD52*-complementation group of HR genes, the RecQ helicase *SGS1*, or the structure-specific endonuclease *MUS81*. In addition, the cohesin mutants do not accumulate Rad52p foci [44], which is indicative of increased DNA damage or DNA repair intermediates. These data suggested that cohesin mutations on their own do not lead to DNA double strand breaks or fork collapse, both of which require HR for resolution. Cohesin dysfunction may impact replication fork dynamics, but these events are tolerable in the presence of fork stabilizing proteins. Cohesin may also have a role in modulating fork progression. As vertebrate replicons span between approximately 60 and 140 kb [56], [57] and cohesin is associated, on average, with DNA every 10–20 kb [58]–[61], each fork must theoretically pass several cohesin complexes [36]. One mechanism to prevent fork collapse is to minimize the distance between the leading strand helicase and the polymerase. If the polymerase stalls and the helicase continues to advance, single stranded DNA is exposed and the fork becomes more fragile. Rad61p association with cohesin is thought to induce a closed cohesin conformation that limits fork progression [36]. When the cohesin ring changes to a more restrictive conformation it may prevent helicase and polymerase separation. Support for cohesin moderating fork progression comes from the finding that human SMC1 is phosphorylated by ATR in response to S phase stress [62] and that this modification is required for activation of the replication checkpoint.

### PARP inhibition reduces the viability of cohesin-depleted cells

In higher eukaryotes, additional factors regulate replication fork stability and progression, one example being the family of poly(ADP-ribose) polymerases (PARPs). PARPs play a major role in DNA metabolism including aspects of repair and replication (reviewed in [63]). PARP was recently found to be activated by stalled replication forks and promoted replication fork restart [45]. We found that loss of PARP and PARG orthologs synergized with a mutation in *him-1/SMC1* in *C. elegans* resulting in a decrease in cell and organismal viability. In human cells, depletion of *SMC1*, *SMC3 or SCC1* by siRNA caused sensitivity to the PARP-inhibitor olaparib. Interestingly, this sensitivity was comparable to that observed when we depleted *BRCA1* by siRNA and then treated cells with olaparib. The interaction between multiple cohesin subunits and PARP was found to be cell line independent, suggesting a robust interaction. Recent emerging hypotheses have proposed alternate fork restart pathways where one branch is HR mediated and the other requires PARP [63]. It has been suggested that BRCA1/2 are synthetic lethal with PARP1/2 because of an inability to restart stalled or collapsed forks. This hypothesis is interesting given the connection we observe between cohesin and fork progression mediators.

Given our findings regarding the importance of replication fork mediators in the presence of cohesin mutations, it is possible that PARP activity is needed for replication fork stability or lesion bypass in the cohesin mutants or knockdown cell lines. However, while synthetic lethal interactions between cohesin mutations and replication fork mediators led to testing PARP inhibitors, the mechanism of the PARP-cohesin interaction is not known because PARP and cohesin each have multiple functions that could be co-dependent. For example, it is possible that the synthetic lethality of cohesin knockdown with PARP inhibition is related to the role of cohesin in HR [64] PARP inhibition is known to be effective in killing cells with defective HR, such as *BRCA1*, *BRCA2* and *ATM*-deficient cells [15], [16]. It is possible that the synthetic lethality of cohesin knockdown and PARP inhibition is due to either or both mechanisms. Although further investigation is needed to identify the specific mechanism of lethality, our data indicate PARP inhibition is a potential treatment for tumors with cohesin or cohesin-related mutations, which represent a significant proportion of colorectal, ovarian, breast and other tumors [1]–[3].

## Materials and Methods

### *S. cerevisiae* strain construction and SGA screens

Temperature sensitive (ts) cohesin alleles were marked with *URA3* as described previously [65], [66] and were used as query genes in SGA screens. Query genes were screened against the non-essential deletion collection [31] and a collection of DAmP [33] and ts [32] alleles representing essential genes. SGA screens were performed in biological triplicate, each with three technical replicates using a Singer RoToR essentially as described [67]. *SCC1* and *SCC2* were screened at 30°C while *SMC1* was screened at 25°C due to slow growth at higher temperatures. See Tables S7 and S8 for a full list of *S. cerevisiae* strains used in this study.

### Double mutant reconstruction and random spore analysis

Double heterozygous mutants were recreated by mating each of the single mutants and selecting on −ura, +G418 plates. The cohesin mutant parent strains were the same query genes used for SGA screens and the other parent was pulled directly from the array plates. Heterozygotes were confirmed by PCR analysis to confirm the identity of the array strain. External primers, unique to the upstream and downstream sequences of each gene locus assayed, were used in PCR reactions in most cases. Random spore was performed at 25°C as previously described [30]. Tetrad analysis would have been a true independent measure of a genetic interaction but the cohesin mutants, singly, exhibit relatively high rates of random spore death, making tetrad analysis infeasible on this scale. Briefly, spores were plated onto haploid selection plates containing canavanine and thialysine. Single selection plates were additionally either −ura or +G418. Double mutants that did not grow on the double selection plates (−ura, +G418) were considered SL. Several isolates of viable double mutants were isolated from random spore analysis for growth curve analysis.

### *S. cerevisiae* growth curves

All viable double mutants were analyzed by growth curve analysis as previously described [30] with slight modifications. Briefly, strains were grown overnight in YPD, diluted to an optical density_600 nm_ (OD) of 0.2 and incubated for 4 hours at 25°C. Strains were then diluted to an OD of 0.3 and 100 ul was added to each well of a 96 well plate. 100 ul of fresh YPD was added to each well for a final OD of 0.15. For each plate, fifteen replicates were performed for wild type (WT), while three replicates were performed for each of the other strains analyzed. OD readings were made every 30 min, after 3 min of shaking, over a period of 24 hrs at 26°C (5 plates) or 30°C (6 plates) on a Tecan M1000. Growth curves for each strain can be found in Figure S3.

### Growth curve analysis

Strain fitness was defined as the logarithm of the area under the curve (AUC) and was calculated using Simpson's rule in R [68]. Separate, parallel analyses were performed for plates grown at 26°C and 30°C. Measurements were normalized for plate effects such that the average estimated strain fitness for the wild type curves was constant across each set. Interaction effects were assessed with a linear model of the form:
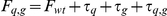


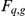
 is the fitness for a double-knockout of query gene 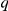 and non-essential gene 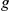, 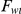 is the fitness for the wild type growth curves, 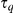 is the single-knockout effect for each of the three query genes (*scc1-73, smc1-259, scc2-4*), 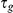 is the single-knockout effect for each of the other non-essential genes assessed, and 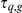 is the double-knockout interaction effect associated with genes 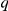 and 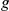. Under additive neutrality, non-interacting gene pairs will have an interaction term 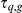 of zero:
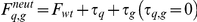
Values of 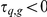 indicate SS interactions, while values of 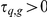 indicate alleviating interactions. After fitting this model, the significance of the estimated interaction effects 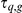 was assessed. See Text S1 and Tables S2, S3, S4 and Figures S4, S5, S6, S7 for additional details and statistical analysis.

### Homolog identification

Homology was determined using protein BLAST to query the *S. cerevisiae* protein sequence against the non-redundant protein sequences database using default parameters. *S. cerevisiae* ORF translation sequences were obtained from the Saccharomyces Genome Database (SGD) and *Homo sapiens* (taxid:9606) and *Caenorhabditis elegans* (taxid:6239) organisms were queried for sequence matches. The algorithm used was blastp (protein-protein BLAST) and all other parameters were set as default settings. Homology was considered to exist if an identified match had an expect value less than 1e-05. In cases where no homologous match was identified in either *C. elegans* or *H. sapiens*, ‘No human, *C. elegans* match’ was entered into Table S1. In some cases, such as with the *S. cerevisiae* gene *DCC1*, no direct *C. elegans* sequence match could be found. However, if the identified *H. sapiens* protein sequence match was used as a BLAST query against the *C. elegans* database a match, *K09H9.2*, could be obtained. In analogous situations, such as with the *S. cerevisiae* gene *MDM20*, the *C. elegans* sequence obtained through BLASTp was used to query the human database for homologous sequences. In these cases if the identified match had an expect value greater than 1e-05 the literature was consulted to determine whether the identified protein was considered homologous by other independent groups. An example where the literature was consulted was to identify the human homolog of *S. cerevisiae RAD61*, *WPL-1*.

### *C. elegans* genetic interactions and SYTO12 staining

The Pvl somatic cell proliferation assay was performed as previously described [30]. SYTO12 staining was performed on young adult worms that had been pre-treated with RNAi by feeding by bleaching gravid adults onto RNAi plates. Worms were incubated in the dark with 33 µM SYTO12 in M9 buffer for 3 hrs and then destained for 1 hour on fresh RNAi plates. Images were captured immediately after destaining on a Zeiss Axioplan 2 microscope using a 40× lens.

### *C. elegans* double mutant strain construction and brood analysis

See Table S9 for strains used. *him-1(e879)* males were mated to either *pme-1(ok988), pme-2(ok344), pme-2(tm3401), pme-3(gk120), pme-4(ok980) or pme-5(ok446)* hermaphrodites. Homozygous *him-1* mutants were followed by the high frequency of males (∼10%) and all other mutations were followed by PCR. *pme-1 him-1* animals were balanced by *hT2* and homozygous *him-1; pme-2(ok344)* animals were balanced by *mIn1*.

### Cell culture and siRNA

Cells were cultured as previously described [14]. siRNAs and transfection reagents were purchased from Dharmacon. For siRNA transfection experiments 300,000 and 550,000 cells were seeded into 6-well dishes for HCT116 and HTB-38 cell lines, respectively. Transfection occurred 24 hours after seeding using ON-TARGETplus 25 nM siRNA pools. Transfection reagent was removed 12 hours post-transfection and following an additional 6–8 hours of recovery cells were transferred to fresh culture dishes and allowed to expand for 3–10 days, depending on the assay. For the clonogenic survival assays 1000 and 500 cells were seeded per well in 24 well dishes and 10 cm plates, respectively. Cells were allowed to attach for 10 hours and then olaparib was added at the indicated concentration. Cells were fixed with 4% paraformaldehyde (PFA) in phosphate buffered saline (PBS) and stained using 0.1% crystal violet in 95% ethanol after 7 and 10 days for the 24 well plate and 10 cm dishes, respectively for HCT116. HTB-38 cells are slower growing and were stained with crystal violet at day 12. Drug exposure was continuous during this time and the 10 cm dishes were supplemented with fresh media containing 0.6 µM olaparib at day 5. High content digital imaging microscopy (HC-DIM) assays were performed by plating 1000 cells per well in 96 well plates. Cells were allowed to settle for 10 hours and then either benzamide or olaparib was added at the indicated concentration. Cells were fixed with PFA after 3 days of drug exposure and stained with Hoescht dye #33258 (Molecular Probes). Plates were subjected to HC-DIM using a Cellomics ArrayScan with a 20× (81 images/well) or 10× (16 images/well) dry lens. The total number of Hoescht-positive nuclei was determined using a Cellomics Target Activation algorithm and normalized to each siRNA treatment (*GAPDH*, *BRCA1* or *SMC1*). Western blots were performed on protein extracts collected from asynchronous, sub-confluent cells harvested 3 days post-transfection as detailed previously [2]. Olaparib was purchased from Selleck and Benzamide was purchased from Aldrich. Antibodies were purchased from Millipore (BRCA1 07-434), Abcam (SMC1 ab9262, alpha Tubulin ab56476) and Trevigen (PAR 4336-BPC-100). HTB-38 and RPE-1 cells were purchased from ATCC and BV79 and BV80 were kindly provided by B. Vogelstein.

## Supporting Information

Figure S1 Cohesin mutations found in colon tumors. Comparison of *SMC1* orthologs in *H. sapiens*, *C. elegans*, and *S. cerevisiae*. Mutations identified in colon tumors are indicated on the human gene and protein domains are shown on the *S. cerevisiae* gene. The number of amino acids (aa) are shown on the right hand side. B) *SCC2* mutations. C) *SCC1* mutations. D) Schematic of cohesin and loaders (adapted from [12]).
(TIF)

Figure S2 SGA network. Negative genetic interactions from three screens were overlaid to find common interactions. Green circles indicate genes conserved in humans and purple circles represent genes with no identifiable sequence orthologs. Circles outlined in black represent essential *S. cerevisiae* genes. 55 genes, not including the cohesin query genes, are represented in this figure.
(TIF)

Figure S3 Growth curve replicates at A) 26°C and B) 30°C. *scc2-4* growth curves were only run at 30°C and *smc1-259* curves were only assayed at 26°C. *scc1-73* curves were run at both temperatures because unlike the other two alleles, *scc1-73* shows a phenotype at both temperatures. In most cases if an interaction with *scc1-73* was present, it was more pronounced at 30°C. Some interactions were tested with multiple alleles of the same gene. If an interaction was identified the second allele was not always assayed (denoted by black circles). Double mutants that were SL according to random spore could not be analyzed by growth curve analysis and are marked with ‘SL’. Gene A mutations refer to cohesin alleles and gene B mutations refer to genes identified in the SGA screens.
(TIF)

Figure S4 Strain fitness at 26°C ranked by interaction magnitude. Each growth curve is assigned an individual estimate of strain fitness reflecting the area under the curve (AUC) and these are averaged for each strain. A neutral strain fitness estimate is computed for each interaction and represents the theoretical strain fitness of the double mutant under conditions of an additive, non-synergistic genetic interaction. Synergistic interactions occur when the experimental double mutant strain fitness deviates from the neutral estimate. Interactions are ranked according to the difference between the experimental and neutral strain fitness estimates with stronger negative interactions occurring at the top of the figure.
(TIF)

Figure S5 Strain fitness at 30°C ranked by interaction magnitude. See Figure S4 legend for additional details.
(TIF)

Figure S6 Interaction T-statistics (26°C). T-statistics are ranked according to magnitude. Dotted lines indicate a p-value cut off of .05, and dashed lines indicate a Bonferroni corrected p-value of .05.
(TIF)

Figure S7 Interaction T-statistics (30°C). T-statistics are ranked according to magnitude. Dotted lines indicate a p-value cut off of .05, and dashed lines indicate a Bonferroni corrected p-value of .05.
(TIF)

Figure S8 Replicative stress and increased apoptosis is seen in him-1 mutants when treated with RNAi against genes that mediate replication fork progression. A) Graph showing the average number of apoptotic corpses per gonad arm. Predicted bars represent the sum of the background levels of apoptotic corpses in WT and him-1 and the effect of the RNAi on WT. B) Representative images showing apoptotic corpses in untreated WT and him-1 worms and worms treated with *RAD61*, *CSM3*, and *CTF8* RNAi. Error bars represent SEM.
(TIF)

Figure S9 Knockout of *RAD51* does not rescue the lethality of cohesion, fork mediator double mutants. *RAD51* was replaced with the resistance gene for hygromycin in double heterozygous *smc1-259* and fork mediator (*CSM3, CTF4, CTF8, DCC1, RAD61*) mutants. The same was done for scc1-73, fork mediator double mutants. Random spore was performed on all 10 triple mutants and no rescue of lethality was seen in any cases. Random spore results are shown for A) *scc1-73, csm3Δ, rad51Δ* and B) *smc1-259, ctf8Δ, rad51Δ* triple heterozygotes.
(TIF)

Figure S10 HCT116 cells treated with siRNAs targeting various cohesion genes are sensitive to the PARP inhibitor Benzamide. HCT116 cells were transfected with the siRNA indicated and exposed to 5 mM Benzamide for 3 days before fixing, staining with Hoescht, and counting cell number using HC-DIM. Error bars represent SEM.
(TIF)

Table S1 *S. cerevisiae*, *C. elegans* and human orthologs.
(DOCX)

Table S2 Double mutant interactions ranked by T-statistic at 26°C.
(DOCX)

Table S3 Double mutant interactions ranked T-statistic at 30°C.
(DOCX)

Table S4 Summary of interactions identified by Growth Curve Analysis.
(DOCX)

Table S5 Reported CIN Phenotypes of cohesin interacting genes.
(DOCX)

Table S6 SGA scores for the major genes involved in homologous recombination.
(DOCX)

Table S7 Diploid *S. cerevisiae* strains used in this study.
(DOCX)

Table S8 Haploid *S. cerevisiae* strains used in this study.
(DOCX)

Table S9 *C. elegans* strains.
(DOCX)

Text S1 Supplementary methods.
(DOCX)

## References

[1] Xu H, Yan M, Patra J, Natrajan R, Yan Y (2011). Enhanced RAD21 cohesin expression confers poor prognosis and resistance to chemotherapy in high grade luminal, basal and HER2 breast cancers.. Breast Cancer Res.

[2] Barber TD, McManus K, Yuen KW, Reis M, Parmigiani G (2008). Chromatid cohesion defects may underlie chromosome instability in human colorectal cancers.. Proc Natl Acad Sci U S A.

[3] Solomon DA, Kim T, Diaz-Martinez LA, Fair J, Elkahloun AG (2011). Mutational inactivation of STAG2 causes aneuploidy in human cancer.. Science.

[4] Oikawa K, Ohbayashi T, Kiyono T, Nishi H, Isaka K (2004). Expression of a novel human gene, human wings apart-like (hWAPL), is associated with cervical carcinogenesis and tumor progression.. Cancer Res.

[5] Ghiselli G, Iozzo RV (2000). Overexpression of bamacan/SMC3 causes transformation.. J Biol Chem.

[6] Zhang N, Ge G, Meyer R, Sethi S, Basu D (2008). Overexpression of separase induces aneuploidy and mammary tumorigenesis.. Proc Natl Acad Sci U S A.

[7] Hagemann C, Weigelin B, Schommer S, Schulze M, Al-Jomah N (2011). The cohesin-interacting protein, precocious dissociation of sisters 5A/sister chromatid cohesion protein 112, is up-regulated in human astrocytic tumors.. Int J Mol Med.

[8] Sheltzer JM, Blank HM, Pfau SJ, Tange Y, George BM (2011). Aneuploidy drives genomic instability in yeast.. Science.

[9] Michaelis C, Ciosk R, Nasmyth K (1997). Cohesins: Chromosomal proteins that prevent premature separation of sister chromatids.. Cell.

[10] Uhlmann F, Lottspeich F, Nasmyth K (1999). Sister-chromatid separation at anaphase onset is promoted by cleavage of the cohesin subunit Scc1.. Nature.

[11] Uhlmann F, Wernic D, Poupart MA, Koonin EV, Nasmyth K (2000). Cleavage of cohesin by the CD clan protease separin triggers anaphase in yeast.. Cell.

[12] Nasmyth K, Haering CH (2009). Cohesin: Its roles and mechanisms.. Ann Rev Genet.

[13] Hartwell LH, Szankasi P, Roberts CJ, Murray AW, Friend SH (1997). Integrating genetic approaches into the discovery of anticancer drugs.. Science.

[14] McManus K, Barrett I, Nouhi Y, Hieter P (2009). Specific synthetic lethal killing of RAD54B-deficient human colorectal cancer cells by FEN1 silencing.. PNAS.

[15] Bryant HE, Schultz N, Thomas HD, Parker KM, Flower D (2005). Specific killing of BRCA2-deficient tumors with inhibitors of poly(ADP-ribose) polymerase.. Nature.

[16] Farmer H, McCabe N, Lord CJ, Tutt AN, Johnson DA (2005). Targeting the DNA repair defect in BRCA mutant cells as a therapeutic strategy.. Nature.

[17] Audeh MW, Carmichael J, Penson RT, Friedlander M, Powell B (2010). Oral poly(ADP-ribose) polymerase inhibitor olaparib in patients with BRCA1 or BRCA2 mutations and recurrent ovarian cancer: A proof-of-concept trial.. Lancet.

[18] O'Shaughnessy J, Osborne C, Pippen JE, Yoffe M, Patt D (2011). Iniparib plus chemotherapy in metastatic triple-negative breast cancer.. N Engl J Med.

[19] Khan OA, Gore M, Lorigan P, Stone J, Greystoke A (2011). A phase I study of the safety and tolerability of olaparib (AZD2281, KU0059436) and dacarbazine in patients with advanced solid tumors.. Br J Cancer.

[20] Tutt A, Robson M, Garber JE, Domchek SM, Audeh MW (2010). Oral poly(ADP-ribose) polymerase inhibitor olaparib in patients with BRCA1 or BRCA2 mutations and advanced breast cancer: A proof-of-concept trial.. Lancet.

[21] Costanzo M, Baryshnikova A, Bellay J, Kim Y, Spear ED (2010). The genetic landscape of a cell.. Science.

[22] Tong AH, Evangelista M, Parsons AB, Xu H, Bader GD (2001). Systematic genetic analysis with ordered arrays of yeast deletion mutants.. Science.

[23] Vogelstein B, Kinzler K (2004). Cancer genes and the pathways they control.. Nat Med.

[24] Collins SR, Miller KM, Maas NL, Roguev A, Fillingham J (2007). Functional dissection of protein complexes involved in yeast chromosome biology using a genetic interaction map.. Nature.

[25] Boulton SJ, Martin JS, Polanowska J, Hill DE, Gartner A (2004). BRCA1/BARD1 orthologs required for DNA repair in *Caenorhabditis elegans*.. Curr Biol.

[26] Martin JS, Winkelmann N, Petalcorin MI, McIlwraith MJ, Boulton SJ (2005). RAD-51-dependent and -independent roles of a *Caenorhabditis elegans* BRCA2-related protein during DNA double-strand break repair.. Mol Cell Biol.

[27] Derry WB, Putzke AP, Rothman JH (2001). *Caenorhabditis elegans* p53: Role in apoptosis, meiosis, and stress resistance.. Science.

[28] Gagnon SN, Hengartner MO, Desnoyers S (2002). The genes *pme-1* and *pme-2* encode two poly(ADP-ribose) polymerases in *Caenorhabditis elegans*.. Biochem J.

[29] Kirienko NV, Mani K, Fay DS (2010). Cancer models in *Caenorhabditis elegans*.. Dev Dyn.

[30] McLellan J, O'Neil N, Tarailo S, Stoepel J, Bryan J (2009). Synthetic lethal genetic interactions that decrease somatic cell proliferation in *Caenorhabditis elegans* identify the alternative RFC CTF18 as a candidate cancer drug target.. Mol Biol Cell.

[31] Winzeler EA, Shoemaker DD, Astromoff A, Liang H, Anderson K (1999). Functional characterization of the *S. cerevisiae* genome by gene deletion and parallel analysis.. Science.

[32] Li Z, Vizeacoumar FJ, Bahr S, Li J, Warringer J (2011). Systematic exploration of essential yeast gene function with temperature-sensitive mutants.. Nat Biotechnol.

[33] Breslow DK, Cameron DM, Collins SR, Schuldiner M, Stewart-Ornstein J (2008). A comprehensive strategy enabling high-resolution functional analysis of the yeast genome.. Nat Methods.

[34] Sulston JE, Horvitz HR (1977). Post-embryonic cell lineages of the nematode, *Caenorhabditis elegans*.. Developmental Biology.

[35] Stirling PC, Bloom MS, Solanki-Patil T, Smith S, Sipahimalani P (2011). The complete spectrum of yeast chromosome instability genes identifies candidate CIN cancer genes and functional roles for ASTRA complex components.. PLoS Genet.

[36] Terret ME, Sherwood R, Rahman S, Qin J, Jallepalli PV (2009). Cohesin acetylation speeds the replication fork.. Nature.

[37] Kueng S, Hegemann B, Peters BH, Lipp JJ, Schleiffer A (2006). Wapl controls the dynamic association of cohesin with chromatin.. Cell.

[38] Sutani T, Kawaguchi T, Kanno R, Itoh T, Shirahige K (2009). Budding yeast Wpl1(Rad61)-Pds5 complex counteracts sister chromatid cohesion-establishing reaction.. Curr Biol.

[39] Skibbens RV (2004). Chl1p, a DNA helicase-like protein in budding yeast, functions in sister-chromatid cohesion.. Genetics.

[40] Tanaka H, Katou Y, Yagura M, Saitoh K, Itoh T (2009). Ctf4 coordinates the progression of helicase and DNA polymerase alpha.. Genes Cells.

[41] Bando M, Katou Y, Komata M, Tanaka H, Itoh T (2009). Csm3, Tof1, and Mrc1 form a heterotrimeric mediator complex that associates with DNA replication forks.. J Biol Chem.

[42] Komata M, Bando M, Araki H, Shirahige K (2009). The direct binding of Mrc1, a checkpoint mediator, to Mcm6, a replication helicase, is essential for the replication checkpoint against methyl methanesulfonate-induced stress.. Mol Cell Biol.

[43] Gartner A, MacQueen AJ, Villeneuve AM (2004). Methods for analyzing checkpoint responses in *Caenorhabditis elegans*.. Methods Mol Biol.

[44] Stirling PC, Chan YA, Minaker SW, Aristizabal MJ, Barrett I (2012). R-loop mediated genome instability in mRNA cleavage and polyadenylation mutants..

[45] Bryant HE, Petermann E, Schultz N, Jemth AS, Loseva O (2009). PARP is activated at stalled forks to mediate Mre11-dependent replication restart and recombination.. EMBO J.

[46] St-Laurent JF, Gagnon SN, Dequen F, Hardy I, Desnoyers S (2007). Altered DNA damage response in *Caenorhabditis elegans* with impaired poly(ADP-ribose) glycohydrolases genes expression.. DNA Repair (Amst).

[47] Gravel C, Stergiou L, Gagnon SN, Desnoyers S (2004). The *C. elegans* gene *pme-5*: Molecular cloning and role in the DNA-damage response of a tankyrase orthologue.. DNA Repair (Amst).

[48] Brattain MG, Fine WD, Khaled FM, Thompson Brattain DE (1981). Heterogeneity of malignant cells from a human colonic carcinoma.. Cancer Res.

[49] Li JH, Zhang J (2001). PARP inhibitors.. IDrugs.

[50] Tentori L, Leonetti C, Scarsella M, Muzi A, Mazzon E (2006). Inhibition of poly(ADP-ribose) polymerase prevents irinotecan-induced intestinal damage and enhances irinotecan/temozolomide efficacy against colon carcinoma.. FASEB J.

[51] Takahashi M, Koi M, Balaguer F, Boland CR, Goel A (2011). MSH3 mediates sensitization of colorectal cancer cells to cisplatin, oxaliplatin, and a poly(ADP-ribose) polymerase inhibitor.. J Biol Chem.

[52] Beckouet F, Hu B, Roig MB, Sutani T, Komata M (2010). An Smc3 acetylation cycle is essential for establishment of sister chromatid cohesion.. Mol Cell.

[53] Rowland BD, Roig MB, Nishino T, Kurze A, Uluocak P (2009). Building sister chromatid cohesion: Smc3 acetylation counteracts an antiestablishment activity.. Mol Cell.

[54] Rolef Ben-Shahar T, Heeger S, Lehane C, East P, Flynn H (2008). Eco1-dependent cohesin acetylation during establishment of sister chromatid cohesion.. Science.

[55] Mayer ML, Pot I, Chang M, Xu H, Aneliunas V (2004). Identification of protein complexes required for efficient sister chromatid cohesion.. Mol Biol Cell.

[56] Walter J, Newport JW (1997). Regulation of replicon size in xenopus egg extracts.. Science.

[57] Blumenthal AB, Kriegstein HJ, Hogness DS (1974). The units of DNA replication in drosophila melanogaster chromosomes.. Cold Spring Harb Symp Quant Biol.

[58] Laloraya S, Guacci V, Koshland D (2000). Chromosomal addresses of the cohesin component Mcd1p.. J Cell Biol.

[59] Parelho V, Hadjur S, Spivakov M, Leleu M, Sauer S (2008). Cohesins functionally associate with CTCF on mammalian chromosome arms.. Cell.

[60] Wendt KS, Peters JM (2009). How cohesin and CTCF cooperate in regulating gene expression.. Chromosome Res.

[61] Wendt KS, Yoshida K, Itoh T, Bando M, Koch B (2008). Cohesin mediates transcriptional insulation by CCCTC-binding factor.. Nature.

[62] Kim ST, Xu B, Kastan MB (2002). Involvement of the cohesin protein, Smc1, in atm-dependent and independent responses to DNA damage.. Genes Dev.

[63] Helleday T (2011). The underlying mechanism for the PARP and BRCA synthetic lethality: Clearing up the misunderstandings.. Mol Oncol.

[64] Sjogren C, Nasmyth K (2001). Sister chromatid cohesion is required for postreplicative double-strand break repair in *Saccharomyces cerevisiae*.. Curr Biol.

[65] Ben-Aroya S, Coombes C, Kwok T, O'Donnell KA, Boeke JD (2008). Toward a comprehensive temperature-sensitive mutant repository of the essential genes of *Saccharomyces cerevisiae*.. Mol Cell.

[66] Ben-Aroya S, Pan X, Boeke JD, Hieter P (2010). Making temperature-sensitive mutants.. Methods Enzymol.

[67] Tong AH, Lesage G, Bader GD, Ding H, Xu H (2004). Global mapping of the yeast genetic interaction network.. Science.

[68] R Development Core Team: R Foundation for Statistical Computing. (2008). R: A language and environment for statistical computing..

