# Supplementary figures and images for: Synthetic Lethality of Cohesins with PARPs and Replication Fork Mediators
Source: PLoS Genet. 2012 Mar 8;8(3):e1002574. doi: 10.1371/journal.pgen.1002574 (PMC3297586; doi:10.1371/journal.pgen.1002574)

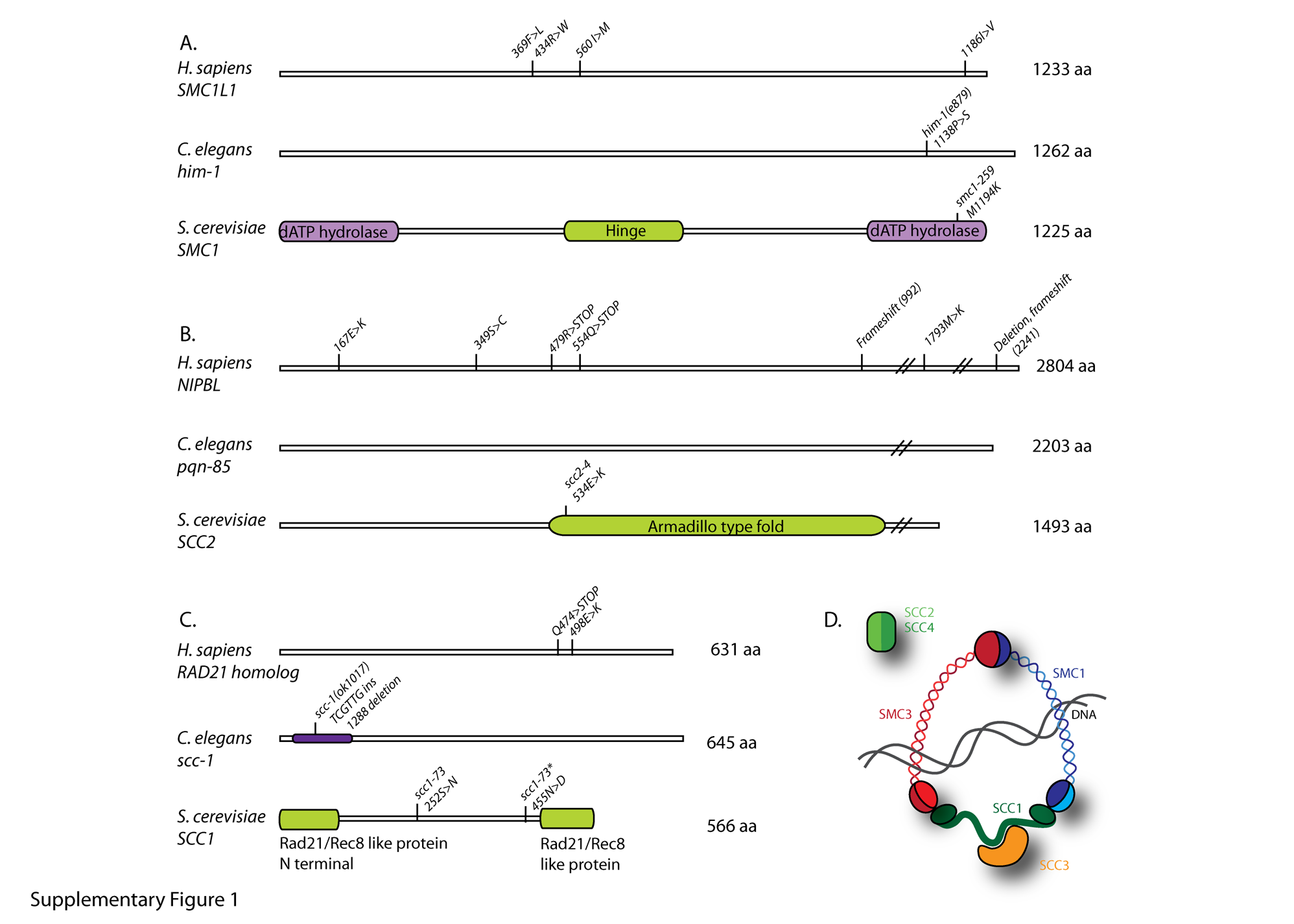

Supplement: Figure S1 — Cohesin mutations found in colon tumors. Comparison of SMC1 orthologs in H. sapiens, C. elegans, and S. cerevisiae. Mutations identified in colon tumors are indicated on the human gene and protein domains are shown on the S. cerevisiae gene. The number of amino acids (aa) are shown on the right hand side. B) SCC2 mutations. C) SCC1 mutations. D) Schematic of cohesin and loaders (adapted from [12]). (TIF) [file pgen.1002574.s001.tif]

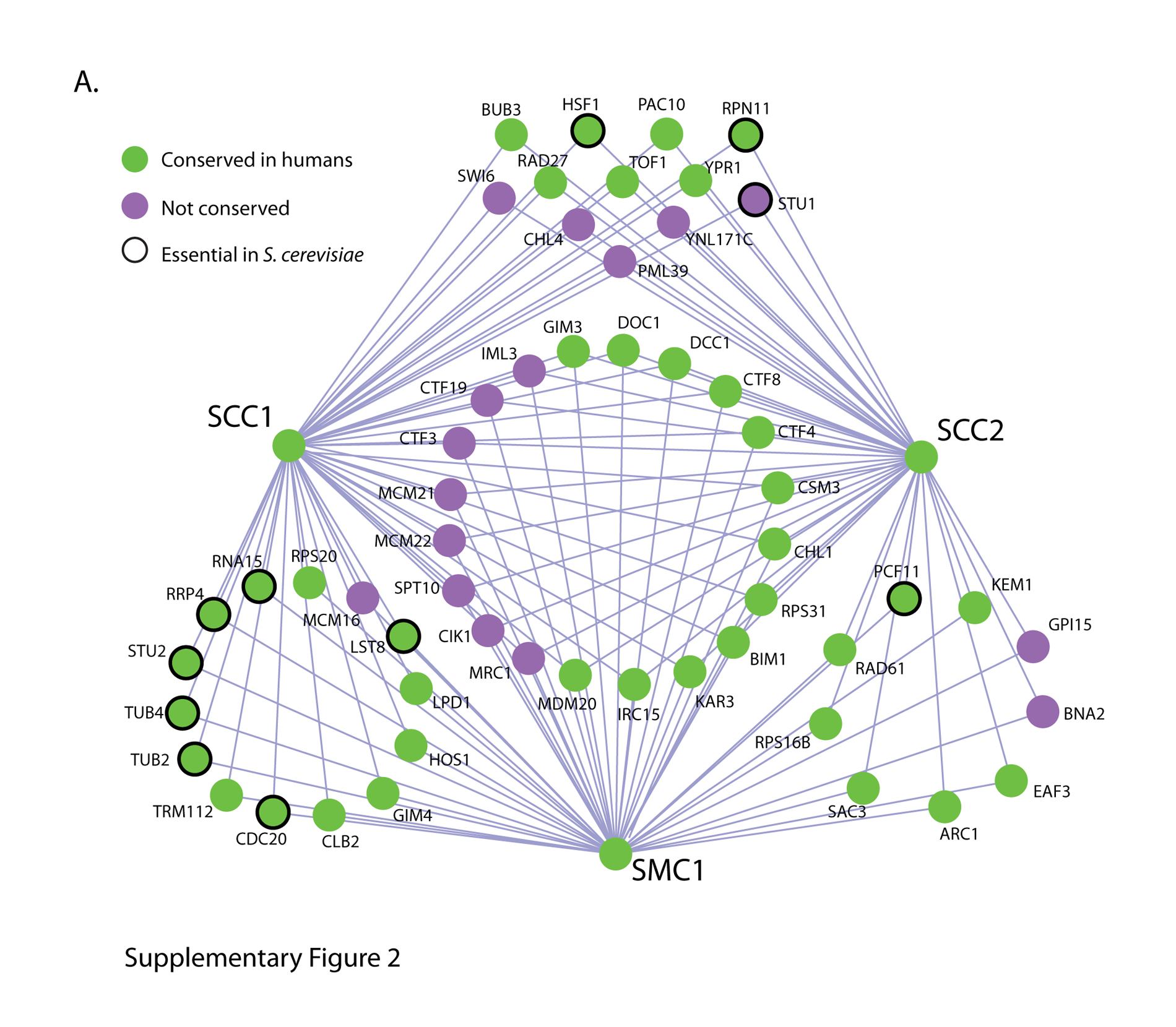

Supplement: Figure S2 — SGA network. Negative genetic interactions from three screens were overlaid to find common interactions. Green circles indicate genes conserved in humans and purple circles represent genes with no identifiable sequence orthologs. Circles outlined in black represent essential S. cerevisiae genes. 55 genes, not including the cohesin query genes, are represented in this figure. (TIF) [file pgen.1002574.s002.tif]

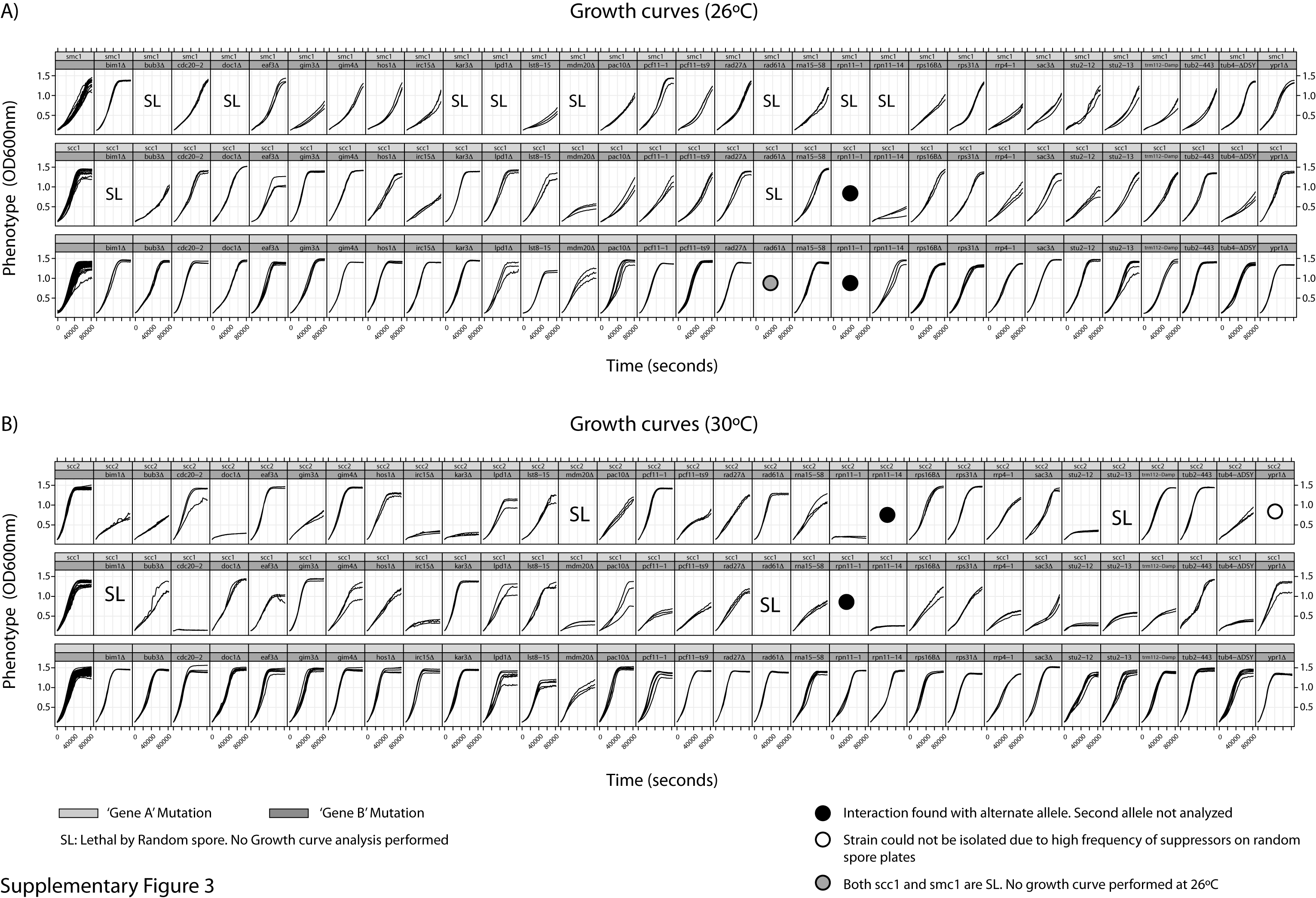

Supplement: Figure S3 — Growth curve replicates at A) 26°C and B) 30°C. scc2-4 growth curves were only run at 30°C and smc1-259 curves were only assayed at 26°C. scc1-73 curves were run at both temperatures because unlike the other two alleles, scc1-73 shows a phenotype at both temperatures. In most cases if an interaction with scc1-73 was present, it was more pronounced at 30°C. Some interactions were tested with multiple alleles of the same gene. If an interaction was identified the second allele was not always assayed (denoted by black circles). Double mutants that were SL according to random spore could not be analyzed by growth curve analysis and are marked with ‘SL’. Gene A mutations refer to cohesin alleles and gene B mutations refer to genes identified in the SGA screens. (TIF) [file pgen.1002574.s003.tif]

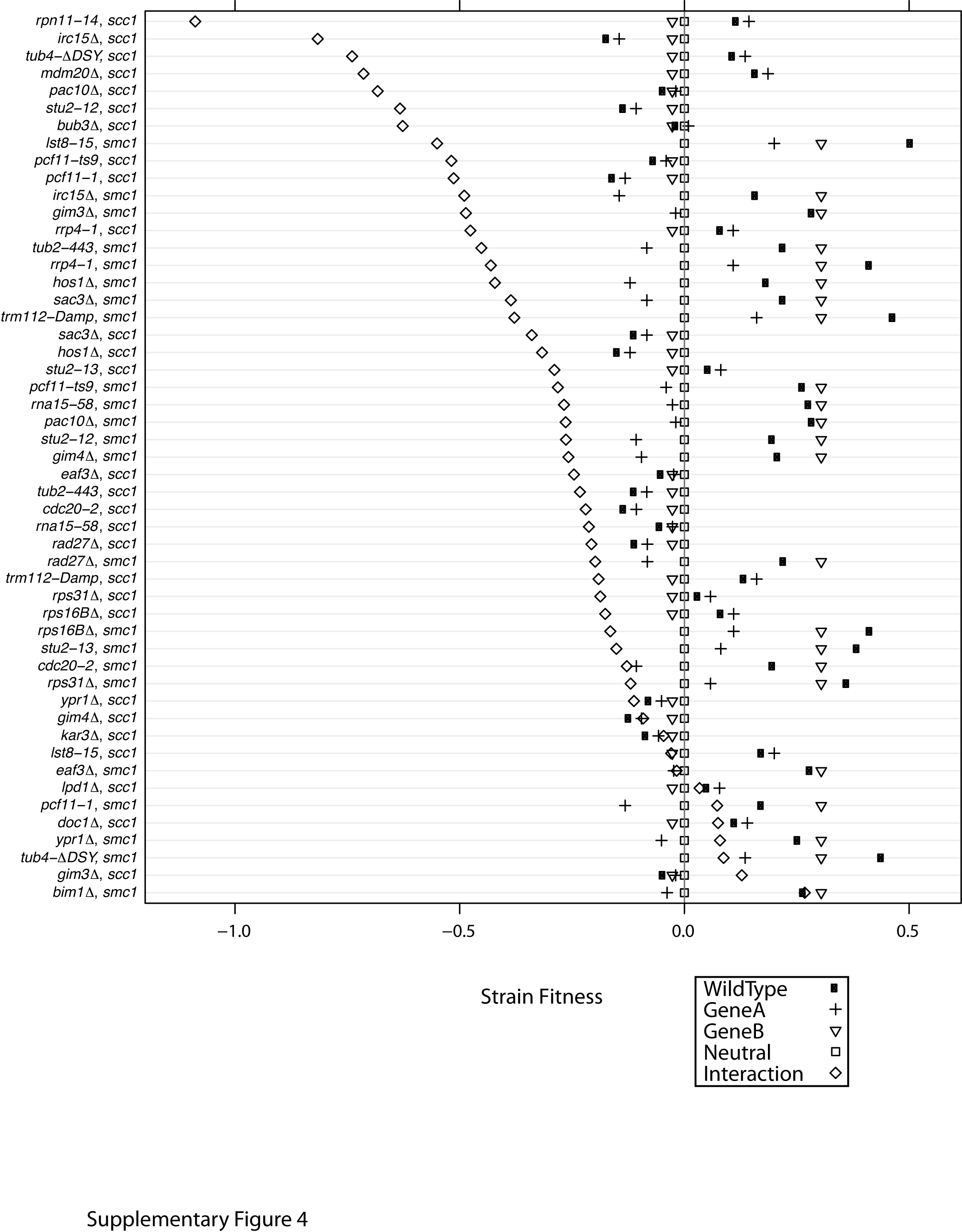

Supplement: Figure S4 — Strain fitness at 26°C ranked by interaction magnitude. Each growth curve is assigned an individual estimate of strain fitness reflecting the area under the curve (AUC) and these are averaged for each strain. A neutral strain fitness estimate is computed for each interaction and represents the theoretical strain fitness of the double mutant under conditions of an additive, non-synergistic genetic interaction. Synergistic interactions occur when the experimental double mutant strain fitness deviates from the neutral estimate. Interactions are ranked according to the difference between the experimental and neutral strain fitness estimates with stronger negative interactions occurring at the top of the figure. (TIF) [file pgen.1002574.s004.tif]

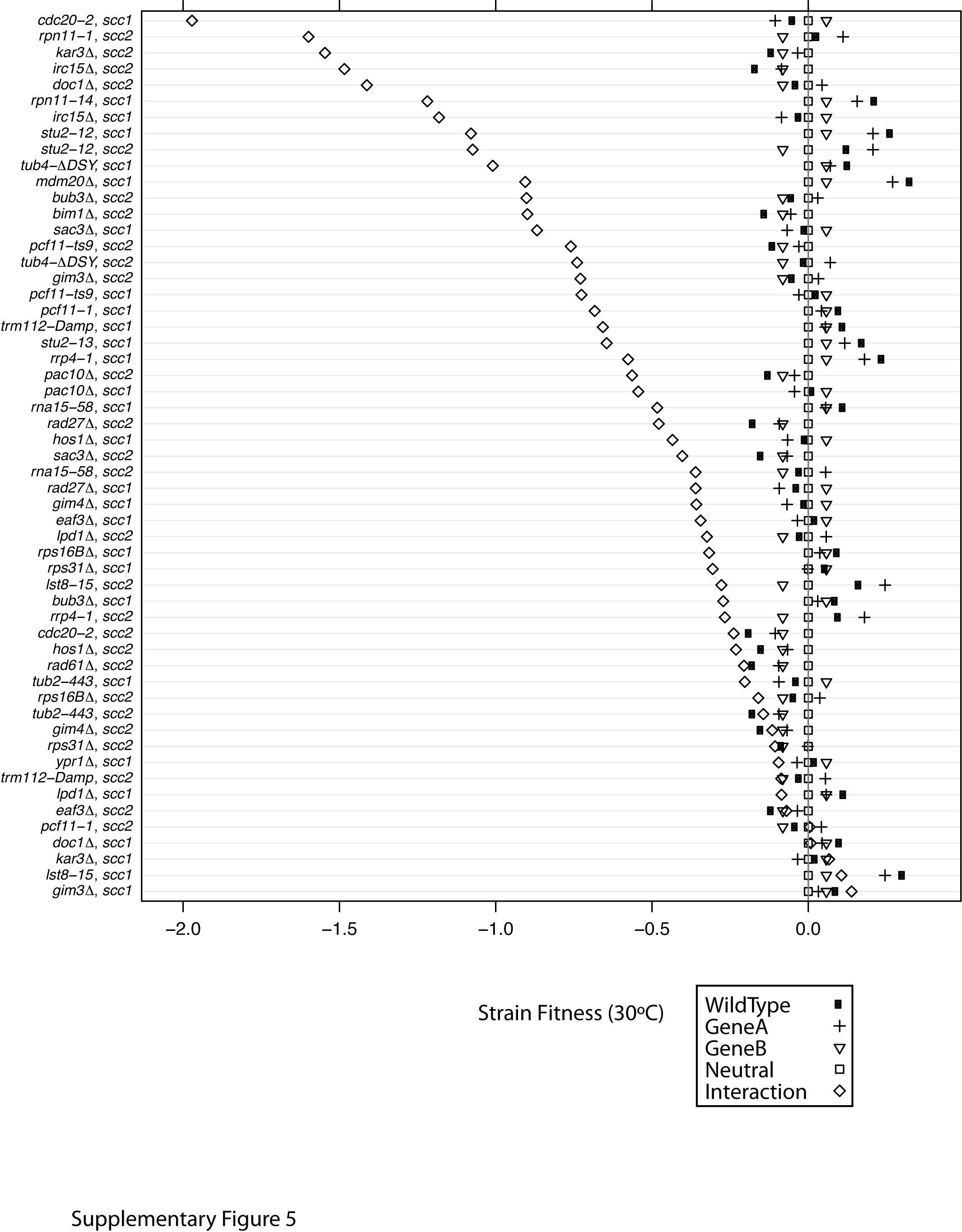

Supplement: Figure S5 — Strain fitness at 30°C ranked by interaction magnitude. See Figure S4 legend for additional details. (TIF) [file pgen.1002574.s005.tif]

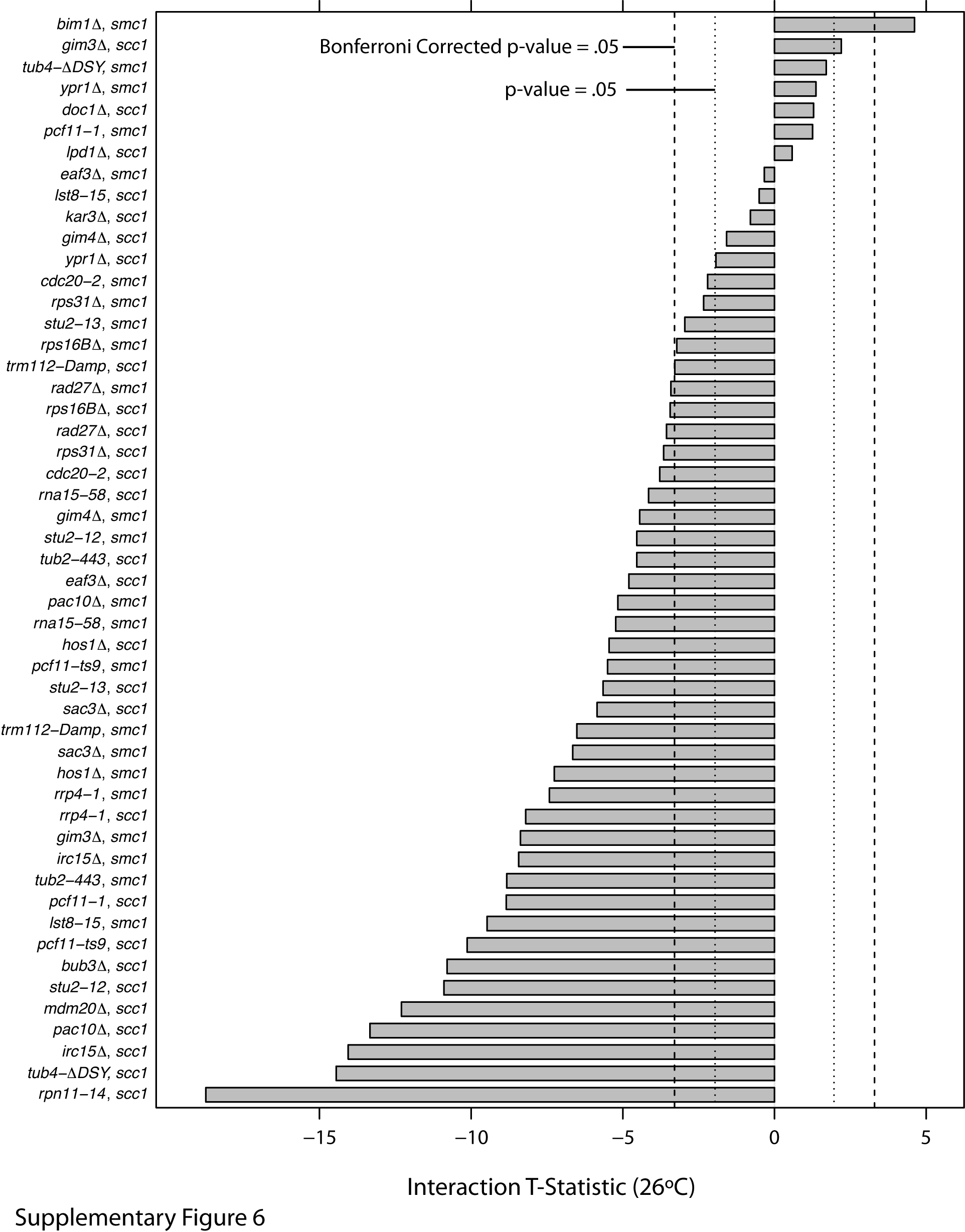

Supplement: Figure S6 — Interaction T-statistics (26°C). T-statistics are ranked according to magnitude. Dotted lines indicate a p-value cut off of .05, and dashed lines indicate a Bonferroni corrected p-value of .05. (TIF) [file pgen.1002574.s006.tif]

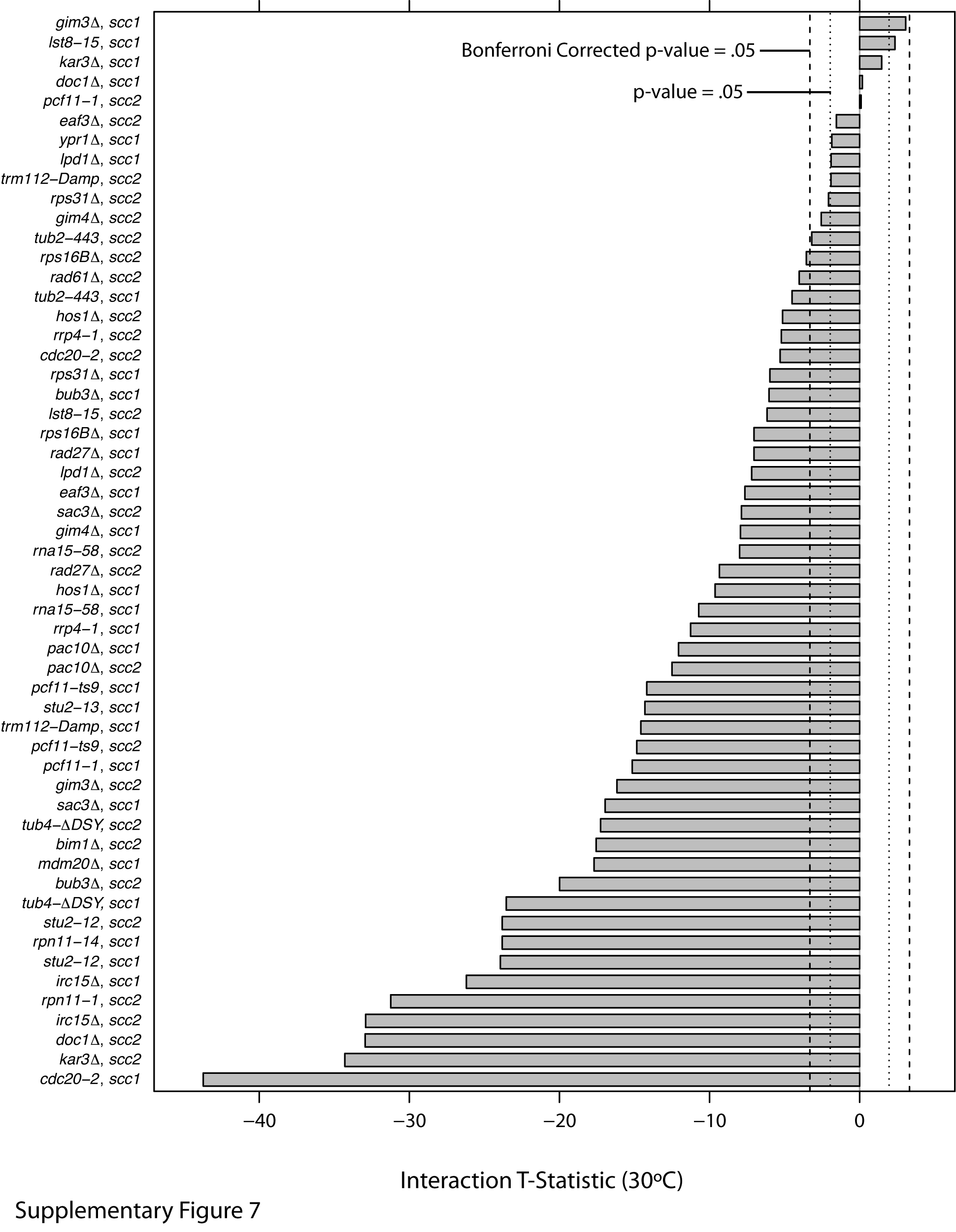

Supplement: Figure S7 — Interaction T-statistics (30°C). T-statistics are ranked according to magnitude. Dotted lines indicate a p-value cut off of .05, and dashed lines indicate a Bonferroni corrected p-value of .05. (TIF) [file pgen.1002574.s007.tif]

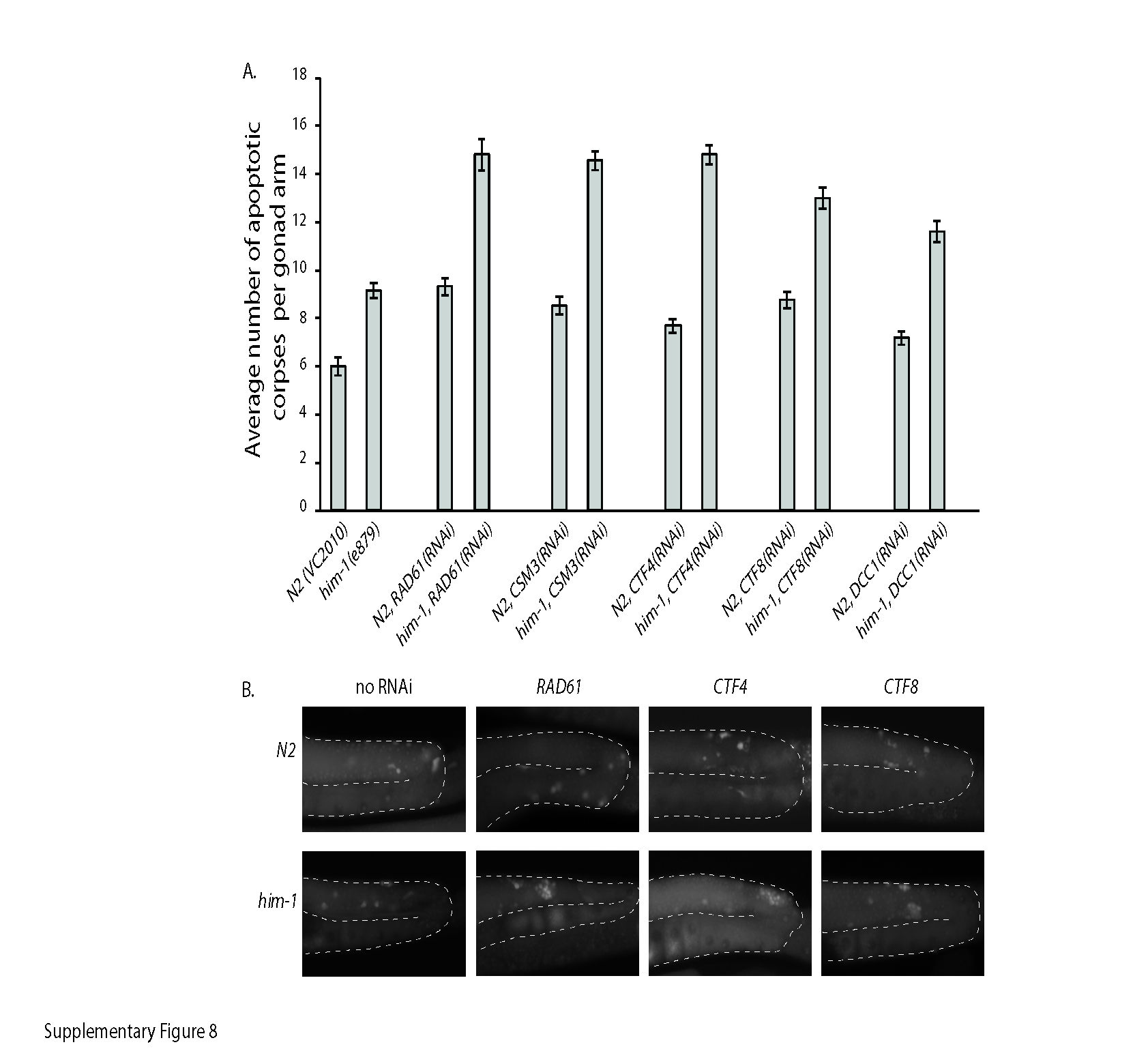

Supplement: Figure S8 — Replicative stress and increased apoptosis is seen in him-1 mutants when treated with RNAi against genes that mediate replication fork progression. A) Graph showing the average number of apoptotic corpses per gonad arm. Predicted bars represent the sum of the background levels of apoptotic corpses in WT and him-1 and the effect of the RNAi on WT. B) Representative images showing apoptotic corpses in untreated WT and him-1 worms and worms treated with RAD61, CSM3, and CTF8 RNAi. Error bars represent SEM. (TIF) [file pgen.1002574.s008.tif]

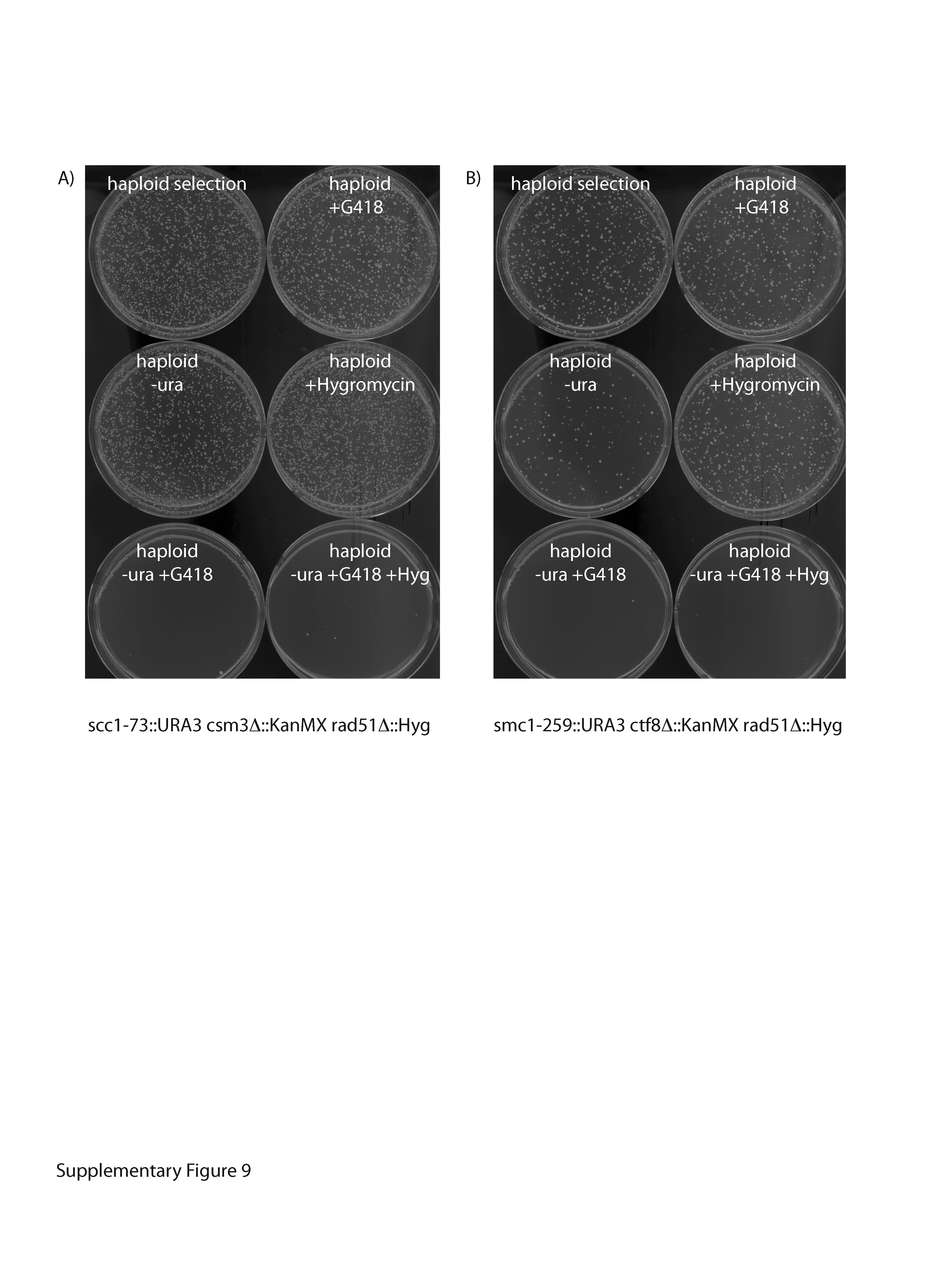

Supplement: Figure S9 — Knockout of RAD51 does not rescue the lethality of cohesion, fork mediator double mutants. RAD51 was replaced with the resistance gene for hygromycin in double heterozygous smc1-259 and fork mediator (CSM3, CTF4, CTF8, DCC1, RAD61) mutants. The same was done for scc1-73, fork mediator double mutants. Random spore was performed on all 10 triple mutants and no rescue of lethality was seen in any cases. Random spore results are shown for A) scc1-73, csm3Δ, rad51Δ and B) smc1-259, ctf8Δ, rad51Δ triple heterozygotes. (TIF) [file pgen.1002574.s009.tif]

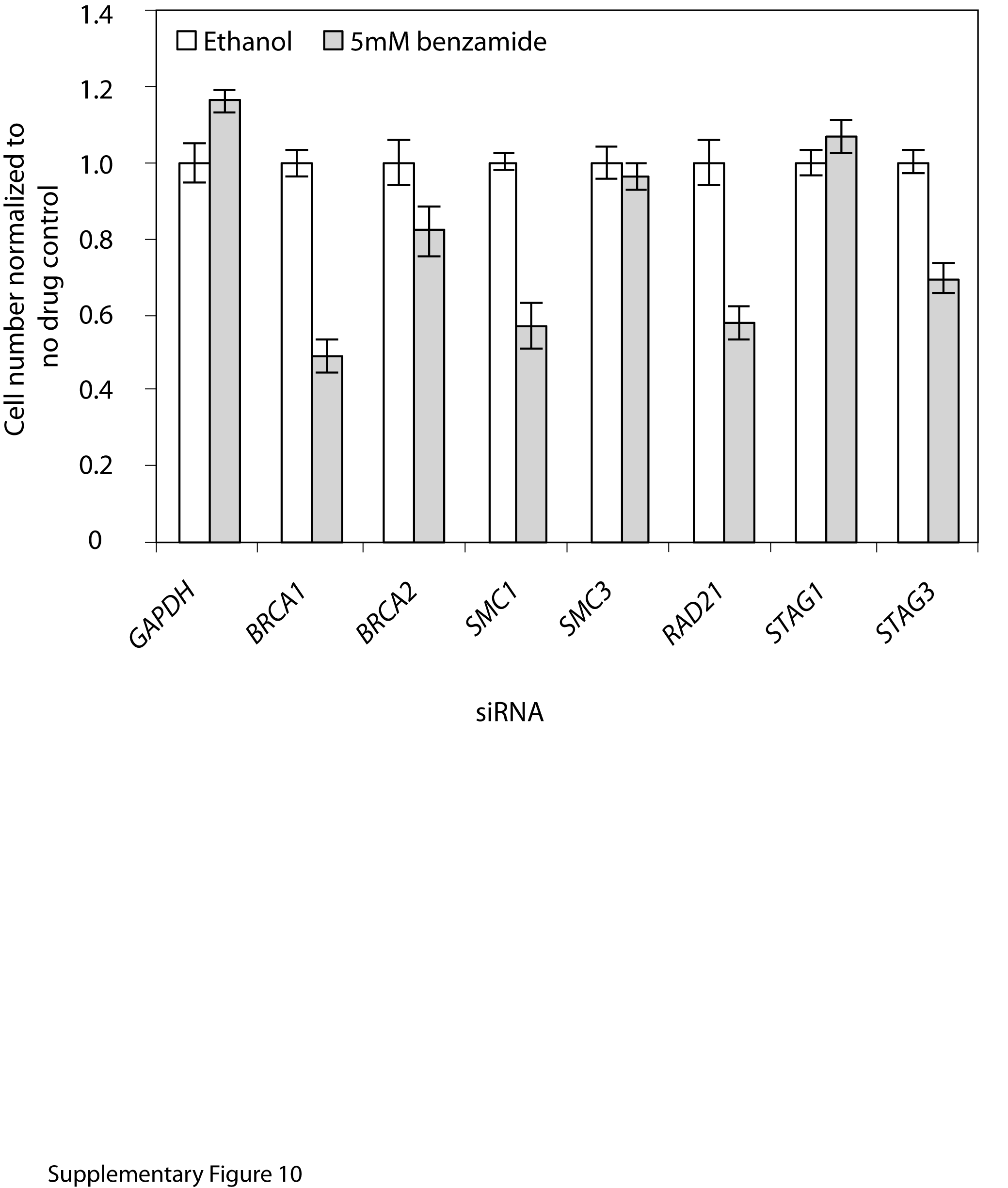

Supplement: Figure S10 — HCT116 cells treated with siRNAs targeting various cohesion genes are sensitive to the PARP inhibitor Benzamide. HCT116 cells were transfected with the siRNA indicated and exposed to 5 mM Benzamide for 3 days before fixing, staining with Hoescht, and counting cell number using HC-DIM. Error bars represent SEM. (TIF) [file pgen.1002574.s010.tif]
