# Supplementary material for: Synthetic Lethality of Cohesins with PARPs and Replication Fork Mediators
Source: PLoS Genet. 2012 Mar 8;8(3):e1002574. doi: 10.1371/journal.pgen.1002574 (PMC3297586; doi:10.1371/journal.pgen.1002574)
Supplement: Table S1 — S. cerevisiae, C. elegans and human orthologs. (DOCX) [file pgen.1002574.s011.docx]

**Table S1:** *S. cerevisiae*, *C. elegans* and human orthologs

| ***S. cerevisiae* Gene**  **(ORF name)** | ***C. elegans* gene**  **(ORF name, e-value)** | ***H. sapiens* gene (e-value)** | ***C. elegans***  **RNAi**  **construct**  **available?** |
| --- | --- | --- | --- |
| *ARC1 (YGL105W)* | *mars-1* (*F58B3.5*, 4E-36) | Aminoacyl tRNA synthase interacting protein (2.00E-39) | Y |
| *BIM1 (YER016W)*^¶^ | *ebp-1* (Y59A8B.7, 1.00E-26)  *ebp-2* (*VW02B12L.3*, 3.00E21) | APC-binding protein EB1 (7.00E-37) | Y |
| *BNA2 (YJR078W)* | No human, *C. elegans* match | | |
| *BUB3 (YOR026W)* | *bub-3* (*Y54G9A.6*, 2.00E-28) | *BUB3* (9.00E-30) | Y |
| *CDC20 (YGL116W)* | *fzy-1* (*ZK177.6*, 8.00E-31) | *CDC20* (3.00E-69) | Y |
| *CHL1 (YPL008W)* | M03C11.2 (9.00E-92) | *DDX11* (2.00E-121) | Y |
| *CHL4 (YDR254W)* | No human, *C. elegans* match | | |
| *CIK1 (YMR198W)* | No human, *C. elegans* match | | |
| *CLB2 (YPR119W)* | *cyb-3* (T06E6.2, 4.00E-26) | *Cyclin B2* (5.00E-55) | Y |
| *CSM3 (YMR048W)* | F23C8.9 (5.00E-06) | TIMELESS interacting protein (8.00E-07) | Y |
| *CTF19 (YPL018W)* | No human, *C. elegans* match | | |
| *CTF3 (YLR381W)* | No human, *C. elegans* match | | |
| *CTF4 (YPR135W)^§^* | *F17C11.10* (1E-53) | WDHD1 (4.00E-18) | Y |
| *CTF8 (YHR191C)^§^* | *T22C1.4* (3E-09) | CHTF8 (5E-10) | Y |
| *DCC1 (YCL016C)^§^* | *K09H9.2* (2E-24) | DCC1 (6.00E-08) | Y |
| *DOC1 (YGL240W)* | *apc-10* (F15H10.3, 3.00E-08) | APC10 (5.00E-22) | Y |
| *EAF3 (YPR023C)* | *mrg-1* (Y37D8A.9, 1.00E-08) | Mortality factor 4-like protein 1 (7.00E-33) | Y |
| *GIM3 (YNL153C)* | *pfd-4* (B0035.4, 2.00E-05) | PFD4 (8.00E-14) | Y |
| *GIM4 (YEL003W)* | *pfd-2* (H20J04.5, 2.00E-05) | PFD2 (4.00E-14) | Y |
| *GPI15 (YNL038W)* | No human, *C. elegans* match | | |
| *HOS1 (YPR068C)* | *hda-1* (C53A5.3, 2.00E-43) | HD1 (3.00E-44) | Y |
| *HSF1 (YGL073W)* | *hsf-1* (Y53C10A.12, 3.00E-19) | HSF1 (3.00E-30) | Y |
| *IML3 (YBR107C)* | No human, *C. elegans* match | | |
| *IRC15 (YPL017C)* | *LLC1.3* (9.00E-73) | Dihydrolipoyl dehydrogenase (2.00E-71) | Y |
| *KAR3 (YPR141C)* | *klp-17* (W02B12.7, 9.00E-53) | Kinesin family member C1  (2.00E-84) | Y |
| *KEM1 (YGL173C)* | *xrn-1* (Y39G8C.1, 4.60E-174) | 5'-3' exoribonuclease 1 (0.00E+00) | Y |
| *LPD1 (YFL018C)* | LLC1.3 (9.00E-160) | Dihydrolipoyl dehydrogenase (4.00E-157) | Y |
| *LST8 (YNL006W)* | *C10H11.8* (3.00E-30) | *LST8* (4.00E-82) | Y |
| *MCM16 (YPR046W)* | No human, *C. elegans* match | | |
| *MCM21 (YDR318W)* | No human, *C. elegans* match | | |
| *MCM22 (YJR135C)* | No human, *C. elegans* match | | |
| *MDM20 (YOL076W)** | *cra-1* (R13F6.10, 0.14) | N(alpha)-acetyltransferase 25 (1.00E-51) | Y |
| *MRC1 (YCL061C)* | No human, *C. elegans* match | | |
| *PAC10 (YGR078C)* | *pfd-3* (T06G6.9, 4.00E-22) | *PFD3* (4.00E-29) | Y |
| *PCF11 (YDR228C)* | *pcf-11* (R144.2, 3.00E-10) | *PCF11* (5.00E-16) | Y |
| *PML39 (YML107C)* | No human, *C. elegans* match | | |
| *RAD27 (YKL113C)* | *crn-1* (Y47G6A.8, 1.00E-112) | *FEN1* (4.00E-118) | Y |
| *RAD61 (YDR014W)^§^* | *wpl-1* (R08C7.10, 1.00E-53) | Wings apart-like protein homolog (7.9) | Y |
| *RNA15 (YGL044C)* | *cpf-2* (F56A8.6, 3.00E-17) | cleavage stimulation factor subunit 2 (6.00E-17) | Y |
| *RPN11 (YFR004W)* | *rpn-11* (K07D4.3, 2.00E-110) | 26S proteasome non-ATPase regulatory subunit 14  (1.00E-115) | Y |
| *RPS16B (YDL083C)* | *rps-16* (T01C3.6, 2.00E-55) | 40S ribosomal protein S16  (9.00E-51) | Y |
| *RPS20 (YHL015W)* | *rps-20* (Y105E8A.16, 3.00E-31) | 40S ribosomal protein S20 (4.00E-32) | Y |
| *RPS31 9YLR167W)* | *ubl-1* (H06I04.4, 7.00E-39) | ubiquitin-40S ribosomal protein S27a precursor (7.00E-59) | N |
| *RRP4 (YHR069C)* | *exos-2* (Y73B6BL.3, 3.00E-43) | *RRP4* (2.00E-56) | N |
| *SAC3 (YDR159W)* | *F20D12.2* (1.00E-26) | MCM3-associated protein (7.00E-33) | Y |
| *SPT10 (YJL127C)* | No human, *C. elegans* match | | |
| *STU1 (YBL034C)* | No human, *C. elegans* match | | |
| *STU2 (YLR045C)* | *zyg-9* (F22B5.7, 4.00E-13) | cytoskeleton-associated protein 5 (3.00E-32) | Y |
| *SWI6 (YLR182W)* | No human, *C. elegans* match | | |
| *TOF1 (YNL273W)^§^* | *tim-1* (Y75B8A.22, 4.00E-66) | timeless homolog (2.00E-09) | Y |
| *TRM112 (YNR046W)* | C04H5.1 (6.00E-10) | tRNA methyltransferase 112 homolog (4.00E-12) | Y |
| *TUB2 (YFL037W)* | *tbb-4* (B0272.1, 0.00E+00) | Beta tubulin (0.00E+00) | Y |
| *TUB4 (YLR212C)* | *tbg-1* (F58A4.8, 9.00E-55) | Gamma tubulin (7.00E-98) | Y |
| *YNL171C* | dubious ORF in S. cerevisiae. | | |
| *YPR1 (YDR368W)* | *Y39G8B.1* (4.00E-49) | aldo-keto reductase family 1, member A1 (9.00E-56) | Y |

^¶^No RNAi construct was available for *ebp-1*. Experiments were performed with RNAi against the second best BLAST match, *ebp-2*.

*No direct *S. cerevisiae* to human match. Used *C. elegans* gene sequence to find a human homolog. Reported e-value for the human gene is from a BLAST search from *C. elegans* against the human database.

^§^No direct *S. cerevisiae* to *C. elegans* match. Used the human gene sequence to find a *C. elegans* homolog. Reported e-value for the *C. elegans* gene is from a BLAST search from human against the *C. elegans* database.
