# Supplementary material for: Synthetic Lethality of Cohesins with PARPs and Replication Fork Mediators
Source: PLoS Genet. 2012 Mar 8;8(3):e1002574. doi: 10.1371/journal.pgen.1002574 (PMC3297586; doi:10.1371/journal.pgen.1002574)
Supplement: Table S2 — Double mutant interactions ranked by T-statistic at 26°C. (DOCX) [file pgen.1002574.s012.docx]

**Table S2:** Double mutant interactions ranked by T-statistic at 26^o^C

| **Rank** | **Double Mutant**^$^ | **Interaction Estimate** | **Standard Error** | **P Value** | **T Statistic** |
| --- | --- | --- | --- | --- | --- |
| 1 | *scc1, rpn11-14* | -1.09 | 0.058 | 7.45E-52 | -18.74 |
| 2 | *scc1, tub4- ΔDSY* | -0.74 | 0.051 | 5.57E-36 | -14.45 |
| 3 | *scc1, irc15Δ* | -0.82 | 0.058 | 1.55E-34 | -14.05 |
| 4 | *scc1, pac10Δ* | -0.68 | 0.051 | 6.06E-32 | -13.34 |
| 5 | *scc1, mdm20Δ* | -0.71 | 0.058 | 3.14E-28 | -12.30 |
| 6 | *scc1, stu2-12* | -0.63 | 0.058 | 2.16E-23 | -10.90 |
| 7 | *scc1, bub3Δ* | -0.63 | 0.058 | 4.81E-23 | -10.79 |
| 8 | *scc1, pcf11-ts9* | -0.52 | 0.051 | 7.75E-21 | -10.13 |
| 9 | *smc1, lst8-15* | -0.55 | 0.058 | 9.98E-19 | -9.48 |
| 10 | *scc1, pcf11-1* | -0.51 | 0.058 | 9.46E-17 | -8.84 |
| 11 | *smc1, tub2-443* | -0.45 | 0.051 | 1.05E-16 | -8.83 |
| 12 | *smc1, irc15Δ* | -0.49 | 0.058 | 1.61E-15 | -8.43 |
| 13 | *smc1, gim3Δ* | -0.49 | 0.058 | 2.45E-15 | -8.37 |
| 14 | *scc1, rrp4-1* | -0.48 | 0.058 | 7.96E-15 | -8.20 |
| 15 | *smc1, rrp4-1* | -0.43 | 0.058 | 1.32E-12 | -7.42 |
| 16 | *smc1, hos1Δ* | -0.42 | 0.058 | 3.60E-12 | -7.26 |
| 17 | *smc1, sac3Δ* | -0.39 | 0.058 | 1.47E-10 | -6.65 |
| 18 | *smc1, trm112-Damp* | -0.38 | 0.058 | 3.18E-10 | -6.52 |
| 19 | *scc1, sac3Δ* | -0.34 | 0.058 | 1.34E-08 | -5.85 |
| 20 | *scc1, stu2-13* | -0.29 | 0.051 | 3.79E-08 | -5.65 |
| 21 | *smc1, pcf11-ts9* | -0.28 | 0.051 | 8.30E-08 | -5.50 |
| 22 | *scc1, hos1Δ* | -0.32 | 0.058 | 1.06E-07 | -5.45 |
| 23 | *smc1, rna15-58* | -0.27 | 0.051 | 3.17E-07 | -5.24 |
| 24 | *smc1, pac10Δ* | -0.26 | 0.051 | 4.54E-07 | -5.16 |
| 25 | *scc1, eaf3Δ* | -0.25 | 0.051 | 2.53E-06 | -4.80 |
| 26 | *scc1, tub2-443* | -0.23 | 0.051 | 8.24E-06 | -4.54 |
| 27 | *smc1, stu2-12* | -0.26 | 0.058 | 8.28E-06 | -4.54 |
| 28 | *smc1, gim4Δ* | -0.26 | 0.058 | 1.27E-05 | -4.44 |
| 29 | *scc1, rna15-58* | -0.21 | 0.051 | 4.35E-05 | -4.15 |
| 30 | *scc1, cdc20-2* | -0.22 | 0.058 | 1.89E-04 | -3.78 |
| 31 | *scc1, rps31Δ* | -0.19 | 0.051 | 3.07E-04 | -3.65 |
| 32 | *scc1, rad27Δ* | -0.21 | 0.058 | 4.32E-04 | -3.56 |
| 33 | *scc1, rps16BΔ* | -0.18 | 0.051 | 6.64E-04 | -3.44 |
| 34 | *smc1, rad27Δ* | -0.20 | 0.058 | 7.27E-04 | -3.42 |
| 35 | *scc1, trm112-Damp* | -0.19 | 0.058 | 1.14E-03 | -3.29 |
| 36 | *smc1, rps16BΔ* | -0.16 | 0.051 | 1.42E-03 | -3.22 |
| 37 | *smc1, stu2-13* | -0.15 | 0.051 | 3.36E-03 | -2.96 |
| 38 | *smc1, rps31Δ* | -0.12 | 0.051 | 2.01E-02 | -2.34 |
| 39 | *smc1, cdc20-2* | -0.13 | 0.058 | 2.81E-02 | -2.21 |
| 40 | *scc1, ypr1Δ* | -0.11 | 0.058 | 5.42E-02 | -1.93 |
| 41 | *scc1, gim4Δ* | -0.09 | 0.058 | 1.16E-01 | -1.58 |
| 42 | *scc1, kar3Δ* | -0.05 | 0.058 | 4.26E-01 | -0.80 |
| 43 | *scc1, lst8-15* | -0.03 | 0.058 | 6.14E-01 | -0.51 |
| 44 | *smc1, eaf3Δ* | -0.02 | 0.051 | 7.38E-01 | -0.34 |
| 45 | *scc1, lpd1Δ* | 0.03 | 0.058 | 5.61E-01 | 0.58 |
| 46 | *smc1, pcf11-1* | 0.07 | 0.058 | 2.10E-01 | 1.26 |
| 47 | *scc1, doc1Δ* | 0.07 | 0.058 | 1.99E-01 | 1.29 |
| 48 | *smc1, ypr1Δ* | 0.08 | 0.058 | 1.73E-01 | 1.37 |
| 49 | *smc1, tub4-ΔDSY* | 0.09 | 0.051 | 8.94E-02 | 1.70 |
| 50 | *scc1, gim3Δ* | 0.13 | 0.058 | 2.86E-02 | 2.20 |
| 51 | *smc1, bim1Δ* | 0.27 | 0.058 | 5.95E-06 | 4.61 |

^$^ Alleles used for *SMC1, SCC1*, and *SCC2* were *smc1-259, scc1-73* and *scc2-4*, respectively.
