# Supplementary material for: Synthetic Lethality of Cohesins with PARPs and Replication Fork Mediators
Source: PLoS Genet. 2012 Mar 8;8(3):e1002574. doi: 10.1371/journal.pgen.1002574 (PMC3297586; doi:10.1371/journal.pgen.1002574)
Supplement: Table S3 — Double mutant interactions ranked T-statistic at 30°C. (DOCX) [file pgen.1002574.s013.docx]

**Table S3:** Double mutant interactions ranked T-statistic at 30^o^C

| **Rank*** | **Double Mutant**^$^ | **Interaction Estimate** | **Standard Error** | **P Value** | **T Statistic** |
| --- | --- | --- | --- | --- | --- |
| 1 | *scc1, cdc20-2* | -1.97 | 0.045 | 5.51E-145 | -43.72 |
| 2 | *scc2, kar3Δ* | -1.55 | 0.045 | 1.06E-114 | -34.29 |
| 3 | *scc2, doc1Δ* | -1.41 | 0.043 | 5.41E-110 | -32.94 |
| 4 | *scc2, irc15Δ* | -1.48 | 0.045 | 7.03E-110 | -32.91 |
| 5 | *scc2, rpn11-1* | -1.60 | 0.051 | 6.73E-104 | -31.24 |
| 6 | *scc1, irc15Δ* | -1.18 | 0.045 | 5.79E-85 | -26.20 |
| 7 | *scc1, stu2-12* | -1.08 | 0.045 | 4.76E-76 | -23.93 |
| 8 | *scc1, rpn11-14* | -1.22 | 0.051 | 1.48E-75 | -23.81 |
| 9 | *scc2, stu2-12* | -1.07 | 0.045 | 1.49E-75 | -23.81 |
| 10 | *scc1, tub4-ΔDSY* | -1.01 | 0.043 | 1.67E-74 | -23.55 |
| 11 | *scc2, bub3Δ* | -0.90 | 0.045 | 4.31E-60 | -19.99 |
| 12 | *scc1, mdm20Δ* | -0.91 | 0.051 | 1.15E-50 | -17.69 |
| 13 | *scc2, bim1Δ* | -0.90 | 0.051 | 4.08E-50 | -17.56 |
| 14 | *scc2, tub4-ΔDSY* | -0.74 | 0.043 | 6.98E-49 | -17.26 |
| 15 | *scc1, sac3Δ* | -0.87 | 0.051 | 1.18E-47 | -16.96 |
| 16 | *scc2, gim3* | -0.73 | 0.045 | 1.88E-44 | -16.17 |
| 17 | *scc1, pcf11-1* | -0.68 | 0.045 | 2.38E-40 | -15.15 |
| 18 | *scc2, pcf11-ts9* | -0.76 | 0.051 | 4.08E-39 | -14.84 |
| 19 | *scc1, trm112-damp* | -0.66 | 0.045 | 4.97E-38 | -14.57 |
| 20 | *scc1, stu2-13* | -0.65 | 0.045 | 5.54E-37 | -14.31 |
| 21 | *scc1, pcf11-ts9* | -0.73 | 0.051 | 1.82E-36 | -14.18 |
| 22 | *scc2, pac10Δ* | -0.56 | 0.045 | 5.71E-30 | -12.50 |
| 23 | *scc1, pac10Δ* | -0.54 | 0.045 | 2.56E-28 | -12.06 |
| 24 | *scc1, rrp4-1* | -0.58 | 0.051 | 2.26E-25 | -11.26 |
| 25 | *scc1, rna15-58* | -0.48 | 0.045 | 2.07E-23 | -10.72 |
| 26 | *scc1, hos1Δ* | -0.43 | 0.045 | 1.15E-19 | -9.63 |
| 27 | *scc2, rad27Δ* | -0.48 | 0.051 | 1.05E-18 | -9.34 |
| 28 | *scc2, rna15-58* | -0.36 | 0.045 | 1.83E-14 | -8.00 |
| 29 | *scc1, gim4Δ* | -0.36 | 0.045 | 2.63E-14 | -7.94 |
| 30 | *scc2, sac3Δ* | -0.40 | 0.051 | 4.19E-14 | -7.88 |
| 31 | *scc1, eaf3Δ* | -0.34 | 0.045 | 2.00E-13 | -7.64 |
| 32 | *scc2, lpd1Δ* | -0.32 | 0.045 | 3.79E-12 | -7.19 |
| 33 | *scc1, rad27Δ* | -0.36 | 0.051 | 1.00E-11 | -7.04 |
| 34 | *scc1, rps16BΔ* | -0.32 | 0.045 | 1.04E-11 | -7.03 |
| 35 | *scc2, lst8-15* | -0.28 | 0.045 | 1.92E-09 | -6.16 |
| 36 | *scc1, bub3Δ* | -0.27 | 0.045 | 4.00E-09 | -6.03 |
| 37 | *scc1, rps31Δ* | -0.31 | 0.051 | 5.47E-09 | -5.98 |
| 38 | *scc2, cdc20-2* | -0.24 | 0.045 | 2.09E-07 | -5.29 |
| 39 | *scc2, rrp4-1* | -0.27 | 0.051 | 3.11E-07 | -5.22 |
| 40 | *scc2, hos1Δ* | -0.23 | 0.045 | 4.69E-07 | -5.13 |
| 41 | *scc1, tub2-443* | -0.20 | 0.045 | 9.00E-06 | -4.51 |
| 42 | *scc2, rad61Δ* | -0.21 | 0.051 | 7.01E-05 | -4.02 |
| 43 | *scc2, rps16BΔ* | -0.16 | 0.045 | 4.41E-04 | -3.55 |
| 44 | *scc2, tub2-443* | -0.14 | 0.045 | 1.55E-03 | -3.19 |
| 45 | *scc2, gim4Δ* | -0.12 | 0.045 | 1.09E-02 | -2.56 |
| 46 | *scc2, rps31Δ* | -0.11 | 0.051 | 3.85E-02 | -2.08 |
| 47 | *scc2, trm112-Damp* | -0.09 | 0.045 | 5.64E-02 | -1.91 |
| 48 | *scc1, lpd1Δ* | -0.09 | 0.045 | 5.81E-02 | -1.90 |
| 49 | *scc1, ypr1Δ* | -0.10 | 0.051 | 6.41E-02 | -1.86 |
| 50 | *scc2, eaf3Δ* | -0.07 | 0.045 | 1.22E-01 | -1.55 |
| 51 | *scc2, pcf11-1* | 0.00 | 0.045 | 9.22E-01 | 0.10 |
| 52 | *scc1, doc1Δ* | 0.01 | 0.043 | 8.55E-01 | 0.18 |
| 53 | *scc1, kar3Δ* | 0.07 | 0.045 | 1.42E-01 | 1.47 |
| 54 | *scc1, lst8-15* | 0.11 | 0.045 | 1.92E-02 | 2.35 |
| 55 | *scc1, gim3Δ* | 0.14 | 0.045 | 2.32E-03 | 3.07 |

^$^ Alleles used for SMC1, SCC1, and SCC2 were *smc1-259, scc1-73* and *scc2-4*, respectively.
