# Supplementary material for: Synthetic Lethality of Cohesins with PARPs and Replication Fork Mediators
Source: PLoS Genet. 2012 Mar 8;8(3):e1002574. doi: 10.1371/journal.pgen.1002574 (PMC3297586; doi:10.1371/journal.pgen.1002574)
Supplement: Table S4 — Summary of interactions identified by Growth Curve Analysis. (DOCX) [file pgen.1002574.s014.docx]

**Table S4:** Summary of interactions identified by Growth Curve Analysis

|  | **# of Potential interactions analyzed** |  | Interaction estimate alone | Interaction  p-value < 0.05 | Bonferroni-corrected  p-value < 0.05 |
| --- | --- | --- | --- | --- | --- |
| 26^o^C | 51 | SS | 44 | 40 | 34 |
|  |  | Alleviating | 7 | 2 | 1 |
|  |  | Total | 51 | 42 | 35 |
| 30^o^C | 55 | SS | 50 | 46 | 43 |
|  |  | Alleviating | 5 | 2 | 0 |
|  |  | Total | 55 | 48 | 43 |

This table does not include SL interactions identified by random spore analysis
