# Supplementary material for: Synthetic Lethality of Cohesins with PARPs and Replication Fork Mediators
Source: PLoS Genet. 2012 Mar 8;8(3):e1002574. doi: 10.1371/journal.pgen.1002574 (PMC3297586; doi:10.1371/journal.pgen.1002574)
Supplement: Table S5 — Reported CIN Phenotypes of cohesin interacting genes. (DOCX) [file pgen.1002574.s015.docx]

**Table S5:** Reported CIN Phenotypes of cohesin interacting genes

| ***S. cerevisiae* Gene** | **CIN phenotypes** |
| --- | --- |
| BIM1 | CTF, BiM, ALF |
| BUB3 | CTF, BiM, ALF |
| CDC20 | CTF |
| CHL1 | CTF, BiM, ALF |
| CSM3 | CTF, BiM, ALF, LOH |
| CTF4 | CTF, BiM, ALF |
| CTF8 | CTF, BiM, ALF, GCR |
| DCC1 | CTF, BiM, ALF, LOH |
| DOC1 | CTF, ALF |
| EAF3 | N/A |
| GIM3 | N/A |
| GIM4 | ALF, BiM |
| HOS1 | CTF (overexpression) |
| IRC15 | CTF, BiM, ALF |
| KAR3 | CTF, ALF |
| LPD1 | N/A |
| LST8 | N/A |
| MDM20 | N/A |
| PAC10 | ALF, BiM |
| PCF11 | CTF |
| RAD27 | CTF, BiM, ALF, LOH, GCR |
| RAD61 | CTF, BiM, ALF |
| RNA15 | CTF |
| RPN11 | CTF |
| RPS16B | N/A |
| RPS31 | N/A |
| RRP4 | CTF, GCR |
| SAC3 | ALF |
| STU2 | N/A |
| TRM112 | N/A |
| TUB2 | Chromosome missegregation |
| TUB4 | CTF |
| YPR1 | N/A |

CTF: chromosome transmission fidelity; BiM: bi-mater; ALF: A-like faker; LOH: loss of heterozygosity; GCR: gross chromosomal rearrangement
