# Supplementary material for: Synthetic Lethality of Cohesins with PARPs and Replication Fork Mediators
Source: PLoS Genet. 2012 Mar 8;8(3):e1002574. doi: 10.1371/journal.pgen.1002574 (PMC3297586; doi:10.1371/journal.pgen.1002574)
Supplement: Table S6 — SGA scores for the major genes involved in homologous recombination. (DOCX) [file pgen.1002574.s016.docx]

**Table S6:** SGA scores for the major genes involved in homologous recombination

| **HR Gene** | **SMC1 screen**  **E-C (p value)** | **SCC1 screen**  **E-C (p value)** | **SCC2 screen**  **E-C (p value)** |
| --- | --- | --- | --- |
| ***RAD51*** | -.027 (.82) | -.006 (.82) | +.13 (.37) |
| ***RAD52*** | -.13 (.70) | -.004 (.96) | -.004 (.97) |
| ***RAD54*** | -.26 (.48) | +.05 (.19) | +.07 (.62) |
| ***RDH54*** | -.14 (.05) | -.02 (.45) | -.01 (.66) |
| ***RAD55*** | +.39 (.10) | -.09 (.01) | +.04 (.30) |
| ***RAD57*** | -.02 (.96) | -.77 (2.5e-14) | -.02 (.83) |
| ***RAD59*** | +.10 (.30) | -.29 (7.2e-5) | -.09 (.08) |
| ***RAD50*** | -.36 (.13) | -.04 (.36) | -.05 (.52) |
| ***MRE11*** | -.39 (.20) | -.16 (.07) | -.12 (.23) |
| ***XRS2*** | -.004 (.98) | -.03 (.72) | +.01 (.81) |
| ***RAD1*** | +.27 (.59) | -.14 (.0002) | -.01 (.72) |
| ***RAD10*** | +.15 (.05) | +.08 (.005) | -.03 (.96) |
| ***RAD9*** | +.13 (.79) | -.005 (.82) | +.06 (.26) |

The grey box highlights the one interaction that would have been considered significant in terms of p value and interaction magnitude in the data filtering process used in this study.
