# Supplementary material for: Synthetic Lethality of Cohesins with PARPs and Replication Fork Mediators
Source: PLoS Genet. 2012 Mar 8;8(3):e1002574. doi: 10.1371/journal.pgen.1002574 (PMC3297586; doi:10.1371/journal.pgen.1002574)
Supplement: Table S7 — Diploid S. cerevisiae strains used in this study. (DOCX) [file pgen.1002574.s017.docx]

**Table S7:** Diploid *S. cerevisiae* strains used in this study

| **Genotype** | **YJM#** | **Genotype** | **YJM#** |
| --- | --- | --- | --- |
| *smc1-259,lpd1Δ* | 591 | *smc1-259, eaf3Δ* | 893 |
| *scc1-73, lpd1Δ* | 592 | *scc1-73, eaf3Δ* | 894 |
| *scc2-4, lpd1Δ* | 635 | *scc2-4, eaf3Δ* | 895 |
| *smc1-259, trm112-Damp* | 636 | *smc1-259, arc1Δ* | 896 |
| *scc1-73, trm112-Damp* | 677 | *scc1-73, arc1Δ* | 897 |
| *scc2-4, trm112-Damp* | 678 | *scc2-4, arc1Δ* | 898 |
| *smc1-259, gim4Δ* | 679 | *smc1-259, sac3Δ* | 899 |
| *scc1-73, gim4Δ* | 680 | *scc1-73, sac3Δ* | 900 |
| *scc2-4, gim4Δ* | 681 | *scc2-4, sac3Δ* | 909 |
| *smc1-259, clb2Δ* | 682 | *smc1-259, pcf11-ts9* | 910 |
| *scc1-73, clb2Δ* | 683 | *scc1-73, pcf11-ts9* | 911 |
| *scc2-4, clb2Δ* | 684 | *scc2-4, pcf11-ts9* | 912 |
| *smc1-259, stu2-12* | 685 | *smc1-259, pcf11-1* | 913 |
| *scc1-73, stu2-12* | 686 | *scc1-73, pcf11-1* | 914 |
| *scc2-4, stu2-12* | 687 | *scc2-4, pcf11-1* | 915 |
| *smc1-259, stu2-13* | 688 | *smc1-259, rad61Δ* | 916 |
| *scc1-73, stu2-13* | 689 | *scc1-73, rad61Δ* | 917 |
| *scc2-4, stu2-13* | 690 | *scc2-4, rad61Δ* | 918 |
| *smc1-259, tub2-443* | 691 | *smc1-259, bim1Δ* | 919 |
| *scc1-73, tub2-443* | 692 | *scc1-73, bim1Δ* | 920 |
| *scc2-4, tub2-443* | 709 | *scc2-4, bim1Δ* | 921 |
| *smc1-259, cdc20-2* | 710 | *smc1-259, rps31-Damp* | 922 |
| *scc1-73, cdc20-2* | 711 | *scc1-73, rps31-Damp* | 923 |
| *scc2-4, cdc20-2* | 712 | *scc2-4, rps31-Damp* | 924 |
| *smc1-259, tub4-ΔDSY* | 713 | *smc1-259, gim3Δ* | 935 |
| *scc1-73, tub4-ΔDSY* | 714 | *scc1-73, gim3Δ* | 936 |
| *scc2-4, tub4-ΔDSY* | 715 | *scc2-4, gim3Δ* | 937 |
| *smc1-259, lst8-15* | 716 | *smc1-259, irc15Δ* | 938 |
| *scc1-73, lst8-15* | 719 | *scc1-73, irc15Δ* | 939 |
| *scc2-4, lst8-15* | 720 | *scc2-4, irc15Δ* | 940 |
| *smc1-259, hos1Δ* | 727 | *smc1-259, kar3Δ* | 941 |
| *scc1-73, hos1Δ* | 728 | *scc1-73, kar3Δ* | 924 |
| *scc2-4, hos1Δ* | 749 | *scc2-4, kar3Δ* | 943 |
| *smc1-259, rrp4-1* | 750 | *smc1-259, doc1Δ* | 944 |
| *scc1-73, rrp4-1* | 751 | *scc1-73, doc1Δ* | 945 |
| *scc2-4, rrp4-1* | 752 | *scc2-4, doc1Δ* | 946 |
| *smc1-259, rna15-58* | 754 | *smc1-259, csm3Δ* | 947 |
| *scc1-73, rna15-58* | 755 | *scc1-73, csm3Δ* | 948 |
| *scc2-4, rna15-58* | 756 | *scc2-4, csm3Δ* | 949 |
| *smc1-259, rpn11-14* | 807 | *smc1-259, mdm20Δ* | 950 |
| *scc1-73, rpn11-14* | 808 | *scc1-73, mdm20Δ* | 951 |
| *scc2-4, rpn11-14* | 813 | *scc2-4, mdm20Δ* | 952 |
| *smc1-259, bub3Δ* | 814 | *smc1-259, chl1Δ* | 953 |
| *scc1-73, bub3Δ* | 815 | *scc1-73, chl1Δ* | 954 |
| *scc2-4, bub3Δ* | 816 | *scc2-4, chl1Δ* | 955 |
| *smc1-259, ypr1Δ* | 817 | *smc1-259, ctf4Δ* | 956 |
| *scc1-73, ypr1Δ* | 818 | *scc1-73, ctf4Δ* | 957 |
| *scc2-4, ypr1Δ* | 819 | *scc2-4, ctf4Δ* | 958 |
| *smc1-259, pac10Δ* | 820 | *smc1-259, rad27Δ* | 959 |
| *scc1-73, pac10Δ* | 825 | *scc1-73, rad27Δ* | 960 |
| *scc2-4, pac10Δ* | 826 | *scc2-4, rad27Δ* | 961 |
| *smc1-259, tof1Δ* | 855 | *smc1-259, dcc1Δ* | 962 |
| *scc1-73, tof1Δ* | 856 | *scc1-73, dcc1Δ* | 963 |
| *scc2-4, tof1Δ* | 863 | *scc2-4, dcc1Δ* | 964 |
| *smc1-259, rps16BΔ* | 864 | *smc1-259, ctf8Δ* | 965 |
| *scc1-73, rps16BΔ* | 879 | *scc1-73, ctf8Δ* | 966 |
| *scc2-4, rps16BΔ* | 880 | *scc2-4, ctf8Δ* | 967 |

All cohesion alleles (*smc1-259, scc1-73, scc2-4*) are marked with *URA3*. All other alleles listed in the table are marked with *KanMX*. In addition to the genotype listed strains are Mat a/alpha ura3*Δ0*/ura3*Δ0* leu2*Δ0/*leu2*Δ0* his3*Δ1*/his3*Δ1* met15*Δ0*/ MET15 or met15*Δ0 LYS2/LYS2* can1*Δ*::STE2pr_pombeHIS5/CAN1 LYP1/lyp1*Δ*
