# Supplementary material for: Synthetic Lethality of Cohesins with PARPs and Replication Fork Mediators
Source: PLoS Genet. 2012 Mar 8;8(3):e1002574. doi: 10.1371/journal.pgen.1002574 (PMC3297586; doi:10.1371/journal.pgen.1002574)
Supplement: Table S8 — Haploid S. cerevisiae strains used in this study. (DOCX) [file pgen.1002574.s018.docx]

**Table S8:** Haploid *S. cerevisiae* strains used in this study

| **Genotype** | **YJM#** | **Genotype** | **YJM#** |
| --- | --- | --- | --- |
| *lpd1Δ* | 589 | *smc1-259, pac10Δ* | 743 |
| *scc1-73, lpd1Δ* | 593 | *scc1-73, pac10Δ* | 745 |
| *scc2-4, lpd1Δ* | 595 | *scc2-4, pac10Δ* | 747 |
| *trm112-Damp* | 597 | *rps16BΔ* | 757 |
| *smc1-259, trm112-Damp* | 599 | *smc1-259, rps16BΔ* | 759 |
| *scc1-73, trm112-Damp* | 601 | *scc1-73, rps16BΔ* | 761 |
| *scc2-4, trm112-Damp* | 603 | *scc2-4, rps16BΔ* | 763 |
| *gim4Δ* | 605 | *eaf3Δ* | 766 |
| *smc1-259, gim4Δ* | 607 | *smc1-259, eaf3Δ* | 767 |
| *scc1-73, gim4Δ* | 609 | *scc1-73, eaf3Δ* | 770 |
| *scc2-4, gim4Δ* | 611 | *scc2-4, eaf3Δ* | 772 |
| *clb2Δ* | 613 | *arc1Δ* | 773 |
| *smc1-259, clb2Δ* | 615 | *smc1-259, arc1Δ* | 775 |
| *scc1-73, clb2Δ* | 617 | *scc1-73, arc1Δ* | 777 |
| *scc2-4, clb2Δ* | 619 | *scc2-4, arc1Δ* | 779 |
| *stu2-12* | 621 | *sac3Δ* | 781 |
| *smc1-259, stu2-12* | 623 | *smc1-259, sac3Δ* | 783 |
| *scc1-73, stu2-12* | 625 | *scc1-73, sac3Δ* | 785 |
| *scc2-4, stu2-12* | 627 | *scc2-4, sac3Δ* | 787 |
| *stu2-13* | 629 | *pcf11-ts9* | 789 |
| *smc1-259, stu2-13* | 631 | *smc1-259, pcf11-ts9* | 791 |
| *scc1-73, stu2-13* | 633 | *scc1-73, pcf11-ts9* | 793 |
| *tub2-443* | 637 | *scc2-4, pcf11-ts9* | 795 |
| *smc1-259, tub2-443* | 639 | *pcf11-1* | 797 |
| *scc1-73, tub2-443* | 641 | *smc1-259, pcf11-1* | 799 |
| *scc2-4, tub2-443* | 643 | *scc1-73, pcf11-1* | 801 |
| *cdc20-2* | 645 | *scc2-4, pcf11-1* | 803 |
| *smc1-259, cdc20-2* | 647 | *rad61Δ* | 805 |
| *scc1-73, cdc20-2* | 649 | *scc2-4, rad61Δ* | 811 |
| *scc2-4, cdc20-2* | 651 | *bim1Δ* | 821 |
| *tub4-ΔDSY* | 653 | *smc1-259, bim1Δ* | 823 |
| *smc1-259, tub4-ΔDSY* | 655 | *scc2-4, bim1Δ* | 827 |
| *scc1-73, tub4-ΔDSY* | 657 | *rps31-Damp* | 829 |
| *scc2-4, tub4-ΔDSY* | 659 | *smc1-259, rps31-Damp* | 831 |
| *lst8-15* | 661 | *scc1-73, rps31-Damp* | 833 |
| *smc1-259, lst8-15* | 663 | *scc2-4, rps31-Damp* | 835 |
| *scc1-73, lst8-15* | 665 | *gim3Δ* | 837 |
| *scc2-4, lst8-15* | 667 | *smc1-259, gim3Δ* | 839 |
| *hos1Δ* | 669 | *scc1-73, gim3Δ* | 841 |
| *smc1-259, hos1Δ* | 671 | *scc2-4, gim3Δ* | 843 |
| *scc1-73, hos1Δ* | 673 | *irc15Δ* | 845 |
| *scc2-4, hos1Δ* | 675 | *smc1-259, irc15Δ* | 847 |
| *rrp4-1* | 693 | *scc1-73, irc15Δ* | 849 |
| *smc1-259, rrp4-1* | 695 | *scc2-4, irc15Δ* | 851 |
| *scc1-73, rrp4-1* | 697 | *kar3Δ* | 853 |
| *scc2-4, rrp4-1* | 699 | *scc1-73, kar3Δ* | 857 |
| *rna15-58* | 701 | *scc2-4, kar3Δ* | 859 |
| *smc1-259, rna15-58* | 703 | *doc1Δ* | 861 |
| *scc1-73, rna15-58* | 705 | *scc1-73, doc1Δ* | 865 |
| *scc2-4, rna15-58* | 707 | *scc2-4, doc1Δ* | 867 |
| *rpn11-14* | 717 | *mdm20Δ* | 877 |
| *scc1-73, rpn11-14* | 721 | *scc1-73, mdm20Δ* | 881 |
| *scc2-4, rpn11-14* | 723 | rad27Δ | 901 |
| *bub3Δ* | 725 | *smc1-259, rad27Δ* | 903 |
| *scc1-73, bub3Δ* | 729 | *scc1-73, rad27Δ* | 905 |
| *scc2-4, bub3Δ* | 731 | *scc2-4, rad27Δ* | 907 |
| *ypr1Δ* | 733 | *smc1-259* | 925 |
| *smc1-259, ypr1Δ* | 735 | *scc1-73* | 927 |
| *scc1-73, ypr1Δ* | 737 | *scc2-4* | 929 |
| *scc2-4, ypr1Δ* | 739 |  | 931 |
| *pac10Δ* | 741 | *Mat alpha scc1-73 | 219 |
| ***Mat alpha *smc1-259* | 561 | *Mat alpha scc2-4 | 212 |

All cohesion alleles (*smc1-259, scc1-73, scc2-4*) are marked with *URA3*. All other alleles listed in the table are marked with *KanMX*. In addition to the genotype listed strains are Mat a ura3*Δ0* leu2*Δ0* his3*Δ1* *MET15 LYS2* can1*Δ*::STE2pr_pombeHIS5 lyp1*Δ*

***SGA query strains. These strains are identical in genotype to the other strains in this table except that they are Mat alpha met15Δ0 or MET15
