# Supplementary material for: Synthetic Lethality of Cohesins with PARPs and Replication Fork Mediators
Source: PLoS Genet. 2012 Mar 8;8(3):e1002574. doi: 10.1371/journal.pgen.1002574 (PMC3297586; doi:10.1371/journal.pgen.1002574)
Supplement: Table S9 — C. elegans strains. (DOCX) [file pgen.1002574.s019.docx]

**Table S9:** *C. elegans* strains

| **Strain Name** | **Genotype** |
| --- | --- |
| CB879 | *him-1(e879) I* |
| RB1042 | *pme-1(ok988) I* |
| VC1171 | *pme-2(ok344) II* |
| FX3401 | *pme-2(tm3401) II* |
| VC130 | *pme-3(gk120) IV* |
| VC641 | *pme-4(ok980) IV* |
| RB684 | *pme-5(ok446) V* |
| PH001 | *pme-1(ok988) I; him-1(e879) I* |
| PH002 | *him-1(e879) I; pme-2(ok344) II* |
| PH003 | *him-1(e879) I; pme-2(tm3401) II* |
| PH004 | *him-1(e879) I; pme-3(gk120) IV* |
| PH005 | *him-1(e879) I; pme-4(ok980) IV* |
| PH006 | *him-1(e879) I; pme-5(ok446)* |
