# Supplementary material for: Synthetic Lethality of Cohesins with PARPs and Replication Fork Mediators
Source: PLoS Genet. 2012 Mar 8;8(3):e1002574. doi: 10.1371/journal.pgen.1002574 (PMC3297586; doi:10.1371/journal.pgen.1002574)
Supplement: Text S1 — Supplementary methods. (DOCX) [file pgen.1002574.s020.docx]

**Supplementary Methods**

**SGA Screens, Scoring and Analysis**

SGA screens were performed essentially as described [1] with slight modifications detailed in Stoepel et al (manuscript in preparation). Briefly, each query strain was mated to the array and split into three biological replicates. Haploids were selected using canavanine, thialysine and minus histidine selection as in [1]. For the final selection step single mutants (array mutants) were selected on plates containing G418 (Invitrogen) and double mutant selection was performed on G418 containing minus uracil plates. For final data collection the three biological replicates were expanded in triplicate for a total of nine replicates and plate images were acquired on a flat bed scanner. Colony size was normalized for each plate and converted to pixel area measurements using custom software developed by the Loewen lab at UBC. Statistical analysis was done in R using a script that compares the colony size measurements for each gene pair (Stoepel et al., manuscript in preparation). The program output is a measure of the difference in colony size, a proxy for strain fitness, of corresponding colonies on the single and double selection plates along with a measure of statistical significance.

**Growth Curve Data Analysis**

The goal of the growth curve analysis of the viable *S. cerevisiae* double mutants was to identify synthetic sick (SS) interactions between three cohesin query genes (*scc1-73, smc1-259, scc2-4*), and 28 of the 33 conserved genes listed in Supplementary Table 1 (the remaining 5 were SL with all three cohesin query genes). Yeast strains were grown for 24 hours on 11 different 96 well plates, each run on a separate day. Of these 11 plates, five were grown at a temperature of 26^o^C, and six at 30^o^C. On each plate there were fifteen replicate wells for the wild type strain and three replicate wells for each of the other strains analyzed. Averaged growth curves for each strain can be found in Figure S3.

Estimation of Strain Fitness

Strain fitness is often defined with implicit reference to exponential growth:

$y\left( t \right)=y_{0}e^{rt}$

where *y(t)* is the population size at time *t*, *y_0_* is the initial population at time *t=0*, and *r* is the exponential growth constant. In reality, unconstrained growth does not exist and it is common to employ a related model, the S-shaped function known as logistic growth: $y\left( t \right)=A+\frac{B-A}{1+exp[-r\left( t-t_{mid} \right)]}$

where *y(t)* and *r* are as defined before. *B* is the carrying capacity or upper asymptote, and *A* is the lower asymptote. *t_mid_* is the time at which population size *y* is halfway between *A* and *B*.

We have successfully employed approaches based on these models previously [2-4] however, in this experiment the heterogeneity of the growth curve shapes prohibits the use of either model. Therefore, we employed a non-parametric method in which we define fitness as the logarithm of the total area under the curve (AUC). We note that this measure is still well-justified in either of the above models and thus represents an attractive strategy that is achievable in practice and compatible with relevant growth models. High values of log(AUC) are associated with large exponential growth constants and hence high strain fitness, and vice versa.

The AUC is calculated for each growth curve through use of Simpson’s rule in R. [5]. After obtaining fitness estimates for each growth curve and prior to identifying SS interactions, it was necessary to address the effect of the difference in temperatures and to normalize for plate (or, equivalently, day) effects. Because the temperature difference had profound effects on the shape of the growth curves, we did separate analyses for plates grown at 26^o^C and 30^o^C. Within each temperature-specific analysis, we exploited the high level of replication for wild type to estimate plate effects and then adjusted all fitness estimates relative to a reference plate (10/07/13 for 26^o^C, 10/06/29 for 30^o^C).

Estimation of Interaction Effects

We performed a derived variable analysis on normalized fitness estimates with a linear model of the form:

$F_{q,g}=F_{wt}+\tau_{q}+ \tau_{g}+ \tau_{q,g}$

$F_{q,g}$ is the fitness for a double-knockout of query gene *q* and non-essential gene *g*, $F_{wt}$ is the fitness for wild type growth curves, $\tau_{q}$ is the single-knockout effect for each of the three query genes (*scc1-73, smc1-259, scc2-4*), $\tau_{g}$ is the single-knockout effect for each of the other non-essential genes assessed, and $\tau_{q,g}$ is the double-knockout interaction effect associated with genes *q* and *g*. After fitting this model, the significance of the interaction effects $\tau_{q,g}$ is assessed by comparing these estimates to those obtained under the assumption of additive neutrality:

$F_{q,g}^{neut}= F_{wt}+ \tau_{q}+ \tau_{g} (\tau_{q,g}=0)$

Hence, values of $\tau_{q,g}<0$ indicate synthetic sick interactions, while values of $\tau_{q,g}>0$ indicate alleviating interactions. See figures S4 and S5 for interaction effects.

Determination of Statistical Significance

To test for the statistical significance of each interaction, T-statistics were calculated as:

$T_{q,g}= \hat{\tau}/SE(\tau_{q,g})$

The T-statistics are the interaction effects normalized according to the standard error of that particular estimate. Bonferroni-corrected p-values were used to control for the family wise error rate (FWER), set at 0.05. Barcharts of the T-statistics associated with these interaction effects are available in Figures S6 and S7, and the estimated values for interaction effects, T-statistics and p-values are listed in Tables S2 and S3. A summary of the interactions analyzed and those found to be statistically significant can be found in Table S4.

**Supplementary References**

1. Tong AH, Evangelista M, Parsons AB, Xu H, Bader GD, et al. (2001) Systematic genetic analysis with ordered arrays of yeast deletion mutants. Science 294: 2364-8.

2.   McLellan J, O'Neil N, Tarailo S, Stoepel J, Bryan J, et al. (2009) Synthetic lethal genetic interactions that decrease somatic cell proliferation in *Caenorhabditis elegans* identify the alternative RFC CTF18 as a candidate cancer drug target. Mol Biol Cell 20(24): 5306-5313.

3. Baetz K, McHardy L, Gable K, Tarling T, Rebérioux D, et al. (2004) Yeast genome-wide drug-induced haploinsufficiency screen to determine drug mode of action. Proc Natl Acad Sci U S A. 101(13): 4525-30.

4. Kennedy MA, Kabbani N, Lambert JP, Swayne LA, Ahmed F, et al. (2011) Srf1 is a novel regulator of phospholipase D activity and is essential to buffer the toxic effects of C16:0 platelet activating factor. PLoS Genet. 7(2):e1001299.

5. R Development Core Team: R Foundation for Statistical Computing. (2008) R: A language and environment for statistical computing.
